# Comparisons of the effects of solute interactions on partition coefficient, k _D_, in selected binary immiscible solvents: a case of oxalic acid and succinic acid

**DOI:** 10.12688/f1000research.55200.1

**Published:** 2022-01-19

**Authors:** Onyeocha V. O., Onuchukwu A. I., Enemo R. E.

**Affiliations:** 1 Department of Chemistry, Federal University of Technology, Owerri, Owerri, Ihiagwa, 1526, Nigeria; 2 Department of Chemistry, CHUKWUEMEKA ODUMEGWU OJUKWU UNIVERSITY, Uli Campus, Anambra, 431124, Nigeria

**Keywords:** Partition Coefficient, Binary Solvent, Oxalic Acid, Succinic Acid, Dimerization, Ionization

## Abstract

**Background:** The molecular distributions of solutes in binary immiscible solvents as used in partition coefficient technique serve as measures of the solute separation, concentration and beneficiation from contaminants.

**Methods:** The effects of solute interactions on partition coefficient, k _D_, in selected binary immiscible solvents were investigated at 30 ^0^C and atmospheric pressure. The activities from the interactions with changes of concentrations within the solvents were analysed. These were done using simple titration method. The solutes were distributed in the binary solvents and the concentrations from the two layers formed were determined by titration method. The interactions of oxalic acid and succinic acid in carbon tetrachloride-water, diethyl ether-water, and n-hexane-water were studied for the partition coefficient values in the respective systems, to determine the nature and degree of the interfering reactions that are affecting the distributions, and to ascertain the best binary solvents from the three systems.

**Results:** Oxalic acid has the partition coefficient of 0.0738 in carbon tetrachloride-water with the dimerization constant of -15.7092 and ionization constant of 0.0303. Oxalic acid has the distribution coefficient of 0.0173, dimerization constant of 144.0167 and the ionization constant of 0.0035 in diethyl ether-water. Oxalic acid has the partition coefficient of 0.0279, dimerization constant of 20.2798 and ionization constant of 0.0019 in n-hexane-water. Succinic acid has the partition coefficient of -0.05617, dimerization constant of -18.5655 and ionization constant of 0.0284 in carbon tetrachloride-water. In diethyl ether-water, succinic acid has the partition coefficient of 0.0427, dimerization constant of -18.1611 and ionization constant of 0.0332. In n-hexane-water, succinic acid has the partition coefficient of -0.04274, dimerization constant of 71.9491 and ionization constant of 0.0265.

**Conclusion:** From these results, carbon tetrachloride-water is recommended for the purification and extraction of oxalic acid from contaminants. Carbon tetrachloride-water is also the best binary immiscible solvent for succinic acid.

## Introduction

When a solute is put in a binary solvent system, the solute circulates itself within the two solvents, until equilibrium is reached, in agreement with Nernst’s distribution law. ^1^ The ratio of the concentrations of the solute in the two layers of the solvents is constant, at any temperature. ^2^^,^^3^ This principle applies on the condition that the solute does not undergo association, or is involved in chemical processes in any of the solvents. ^3^^,^^4^ This partition law is defined by the relation:

$$\frac{C_{X}^{A}}{C_{X}^{B}}=k_{D}$$ (1)

where at equilibrium, $C_{X}^{A}$ and $C_{X}^{B}$ are the relative amounts in moles per litre of the solute in the solvents *A* and *B* respectively, while k _D_ represents the partition coefficient. When the solute is added to the binary solvent and it undergoes a chemical process in one of the liquids or both of them, then the distribution coefficient is affected by the reaction that is occurring in the system. This changes the equation (1) by the expression ^3^:

$$\frac{C_{X}^{A}}{C_{X}^{*B}}=k_{D}+\beta {\left(C_{X}^{*B}\right)}^{y}$$ (2)

where

$$\beta =x{k_{D}}^{x}{\left(\frac{K}{m^{m}n^{n}}\right)}^{\frac{1}{m+n}}$$ (3)

and

$$y=\left(\frac{x}{m+n}\right)-1$$ (4)

$C_{X}^{A}$ is the total amount of the species (X) in moles per litre in the layer *A*; $C_{X}^{*B}$ is the total amount of the species (X) in moles per litre in the layer *B* that was not used in any chemical processes; k _D_ is the parti,tion coefficient; m and n are the stoichiometric coefficients of the products of the dimerization, ionization, or any reaction process happening in solvent *A*; *K* is the dimerization constant (or the reaction constant); and x defines the stoichiometric coefficient of the solute (X) in the solvent.

The constant k _D_ shows the degree of the solubility/dissolution of the solute in the two solvents, *A* and *B*, at the temperature studied. The temperature (T), pH of the solution, and any chemical reaction of the solute with any of the solvents all influence the partition coefficient, k _D_ ( Britannica). ^2^^,^^5^ k _D_ is a fundamental measurable factor that defines the degree of solute recovery in solvent extraction. ^2^^,^^5^ The value of k _D_ depends on the relative solubility of a substance in two-immiscible solvents as expressed by equation (1). The immiscible solvents are chosen based on the physical nature as well as the chemical characteristics of the substance in the binary immiscible solvents. ^2^^,^^7^ The solute when introduced into binary immiscible solvents disperses into the two solvents; when equilibrium reaches, the substance distributes its molecules between the two solvents in line with the Nernst’s distribution law. The ratio of the dissolution of this solute in the binary immiscible solvents is constant at any temperature. ^3^^,^^8^

### Partition law for a chemical reaction system

Consider the equation,

xX+aA⇌mM+nN (5)

In equation (5), the solute, X (for example succinic acid or oxalic acid), disperses into the solvent, A, with the involvement of a chemical reaction to form the products M and N.

When dissociation of the solute X takes place in the solvent A,

m+n>x (6)

So,

$$y<0\;\left(\text{from equation}\;4\right)$$ (7)

and the deviations from simple distribution law is noticed as $C_{X}^{A}$ values tend to zero.

When association of the solute takes place in the solvent,

m+n<x,y>0 (8)

the deviations from the simple distribution law will be noticed at larger values of $C_{X}^{A}$ .

This partition coefficient, k _D_, gives the measure of the dispersion of the solute in the binary solvent, A and B at a defined temperature. The temperature (T) of the system and the chemical reaction of the solute with one or both of the solvents influence the partition coefficient, k _D_. ^3^^,^^9^

Organic compounds such as succinic acid, carbon tetrachloride, acetic acid, oxalic acid, n-hexane, associate to form dimers, and polymers. ^10^^,^^11^ Molecules like succinic acid and oxalic acid ionize in an aqueous solution, forming the conjugate anions as shown in scheme I and II below.

Scheme I: Dissociation/ionization of oxalic acid in aqueous solution

Oxalic acid (HOOCCOOH) ionizes in water to form the oxalate, as shown below:

$$\text{HOOCCOOH}\left(s\right)+H_{2}O\left(l\right)\to {\text{HOOCCOO}}^{-}\left(\mathrm{aq}\right)+H^{+}\left(\mathrm{aq}\right)$$ (9)

$${\text{HOOCCOO}}^{-}\left(\mathrm{aq}\right)+H_{2}O\left(l\right)\to {}^{-}{\text{OOCCOO}}^{-}\left(\mathrm{aq}\right)+H^{+}\left(\mathrm{aq}\right)$$ (10)

$$\left\{\left({}^{-}{\text{OOCCOO}}^{-}\right)\;\text{is the oxalate},C_{2}{O_{4}}^{2-}\right\}$$

The stability of the dimer of oxalic acid has a minimum energy structure that is stabilized by two intermolecular and four intramolecular hydrogen bonds. The most stable structure in previous studies is supported by two intermolecular, and three intramolecular hydrogen bonds. ^12^

Scheme II: Dissociation/ionization of succinic acid

Succinic acid is a dicarboxylic acid also, (HOOCCH _2_CH _2_COOH).

$${\left({\mathrm{CH}}_{2}\right)}_{2}{\left({\mathrm{CO}}_{2}H\right)}_{2}\to {\left({\mathrm{CH}}_{2}\right)}_{2}{{\left({\mathrm{CO}}_{2}\right)}_{2}}^{2-}+{{\text{2H}}_{\mathrm{aq}}}^{+}$$ (11)

Succinic acid could undergo two successive reactions as a diprotic acid. ^10^

Thus,

$${\left({\mathrm{CH}}_{2}\right)}_{2}{\left({\mathrm{CO}}_{2}H\right)}_{2}\to {\left({\mathrm{CH}}_{2}\right)}_{2}\left({\mathrm{CO}}_{2}H\right){\left({\mathrm{CO}}_{2}\right)}^{-}+H^{+}$$ (12)

$${\left({\mathrm{CH}}_{2}\right)}_{2}\left({\mathrm{CO}}_{2}H\right){\left({\mathrm{CO}}_{2}\right)}^{-}\to {\left({\mathrm{CH}}_{2}\right)}_{2}{{\left({\mathrm{CO}}_{2}\right)}_{2}}^{2-}+H^{+}$$ (13)

Succinic acid is fairly soluble in water, and sparingly soluble in ether. On heating succinic acid, a large amount sublimes and the remainder is converted into the cyclic anhydride (succinic anhydride). Succinic acid also gives anhydrides in organic solvents at low temperatures. This is shown below in scheme III.


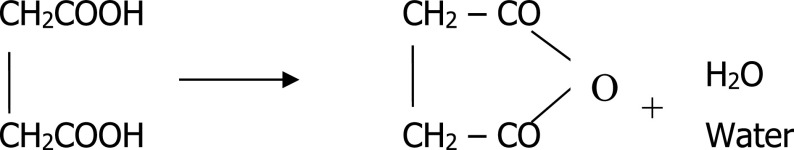

(14)



Scheme III: The succinic anhydride formation of succinic acid.

The degree of the dissolution of non-ionic compounds like succinic acid and oxalic acid is dependent on their polarity. Non-polar compounds dissolve in non-polar solvents while highly polar compounds dissolve in highly polar solvents. A carbon tetrachloride molecule is balanced ionically, while water molecules are involved in the strong hydrogen bonding. Carbon tetrachloride is a non-polar solvent, and water is a polar solvent. Carbon tetrachloride is insoluble in water, this is due to the fact that water molecules are bonded to each other by strong dipole-dipole bonds (hydrogen bonding). ^11^^,^^13^ There are weak attractive forces between water molecules and carbon tetrachloride molecules. ^10^^,^^14^ Carbon tetrachloride is immiscible with water.

The formation of dimers, anhydrides, and ions in the binary immiscible solvents affects the distribution of solutes in the binary immiscible solvents. A high dimerization constant (association constant), K, or ionization constant, α , results in a small value of the partition coefficient, and less efficiency of partition coefficient technique, in solvent extraction as depicted in the equation:

$$\frac{C_{X}^{A}}{C_{X}^{B}}<1$$ (15)

### Solute and solvent interactions

Many compounds and substances such as oxalic acid, acetic acid, ethylenediaminetetraacetic acid and succinic acid can exist in the form of dimers or anhydrides in non-hydrogen bonding liquids. ^10^^,^^14^ Most compounds like oxalic acid and succinic acid are crystals in their pure state. This discussion focuses on a compound with lattice structure. When the crystal is introduced into the solvent, the forces that hold the crystal units together are weakened; consequently, the units are liberated for solvation and mobility. ^15^ According to Onuchukwu, ^15^ ions in a solution devoid of any electric field are known to indulge in identical random movement characterized by random swimming in the solution.

The degrees of the dissolution of non-ionic compounds are influenced by their polarity. These non-ionic compounds are soluble in non-ionic solvents while polar substances dissolve in polar solvents. A non-polar compound like carbon tetrachloride is not soluble in water, a polar solvent. This is due to the fact that water molecules (H _2_O) cling to one another by the strong hydrogen bonding that exists between the oxygen and hydrogen atoms of the water molecule. ^2^^,^^11^^,^^13^ Considering the nature of the bonding between water molecules (H _2_O) and carbon tetrachloride molecules (CCl _4_), it is viewed that there are weak van der Waal forces between the molecules of water and the molecules of carbon tetrachloride. ^16^^,^^17^ Hexane is a non-polar solvent *[* *www.sciencedirect.com* *].* It forms a binary immiscible solvent with water. The interaction of solutes with solvents could give rise to changes that result in the dissolution of the solute in the solvents. With succinic acid, the crystal packing of the molecules dislodges, and the molecules are separated from one another. The spaces between the molecules are filled in with the solvent molecules. ^14^ This explanation defines solvation. ^18^ When the mixture is made up of a homogeneous phase where the constituents cannot be separated from one another by a physical means, a solution is formed. The solvent is present in large excess quantity over the solute. Factors such as temperature, the nature of the solute, and the type of solvent are important in contributing to the dissolution of substances in solvents. ^2^^,^^19^

### Ion – ion interaction

The ion-solvent interaction is anchored on an ion with its neighboring water molecules. The ion is related to its environment. Given an ion of interest such as the oxalate (C _2_O _2_^−4^), surrounded by water molecules and ions, further increase in ionic concentration drives the water molecule to the property of ion-solvent attainment after the ion attract each other. This gives rise to an ion-ion interaction. The ion perceives not only solvent dipoles but also other ions. The mutual interactions between and amongst these ions constitute an essential part of the entire picture of ion–ion interaction which defines electrolytic solution. This ion-ion interaction determines the equilibrium properties of electrolytes and, effects the drift of ions. ^15^^,^^19^

### System of variable composition/solution

The constitution of the phase(s) of a solution is changeable, within a limit. A binary solution is made up of two solvents; a ternary solution is made up of three solvents. These solvents are immiscible, or partially miscible. The degree of the miscibility of the solvents is a continuum. The basic measurable factors for the state of the solution are temperature, pressure and concentration. ^2^^,^^18^ Concentration shows the relative amounts of the solute, in moles per litre, of the solvent.

### Partition function, k _D_ of non-reacting system

When oxalic acid crystals as a solute are shaken up in a mixture of water and carbon tetrachloride as immiscible solvents, for example, it is found that the oxalic acid molecules distribute themselves between the water and carbon tetrachloride layers in such a way that at equilibrium, the ratio of the concentration of oxalic acid in these two phases is constant at any temperature studied. ^2^^,^^8^ This observation is direct evidence of the solute-solvent interaction in accordance with attainment of thermodynamic equilibrium as defined in the ratio of solution of the binary immiscible solvent in the equation below. ^2^^,^^11^^,^^19^

$$\text{In}\frac{a_{B}}{a_{A}}=\text{constant}=k_{D}$$ (16)

Equation 16 is the mathematical expression of Nernst distribution law, which states that a substance distributes itself between two solvents until at equilibrium, where the ratio of the activities of the substance in the two phases remains constant at the given temperature and pressure. Additionally, if the concentrations of the solute in the two phases are expressed and designated by concentrations C, then equation (1) holds. ^2^^,^^17^

The constant k _D_ is called the partition coefficient of the solute between the two solvents. ^19^^,^^20^ The magnitude of k _D_ depends on the nature of the solute and the liquids involved, and the temperature ( LibreTexts). The law is valid provided the solute does not undergo any chemical reactions such as association or dissociation in any of the two immiscible solvents. ^2^^,^^3^ Also, the law does not apply to the total concentrations in the two phases. This partition coefficient, k _D_, measures the miscibility of the solute in the binary immiscible solvent formed by liquid A and liquid B, at any temperature. The temperature (T) of the solution and any chemical reaction of the solute in the solvents affect it.. ^2^^,^^3^

### Distribution law for both dimerization and ionization processes

When dimerization of the solute (X) occurs in one of the liquids, the dimerization reaction is represented as:

$$\text{2X}⇌{\left(X\right)}_{2}$$ (17)

x=2andm=1

The equilibrium constant,

$$K=\frac{\left[{\left(X\right)}_{2}\right]}{{\left[X\right]}^{2}}$$ (18)

Using equations (2), (3), and (4),

$$\frac{C_{X}^{A}}{C_{X}^{*B}}=k_{D}+2k_{D}^{2}KC_{X}^{B}$$ (19)

A plot of $\frac{C_{X}^{A}}{C_{X}^{*B}}\;\mathrm{vs}\;C_{X}^{*B}$ is a straight line with intercept k _D_ and a slope that is equal to $2\;k_{D}^{2}K.$ This will enable the calculation of dimerization constant, K.

When ionization of the solute (X) occurs in one of the liquids, the ionization reaction is represented as:

$$X⇌Y^{+}+Z^{-}$$ (20)

The ionization constant, α, is given by:

$$\alpha =\frac{\left[Y^{+}\right]\left[Z^{-}\right]}{\left[X\right]}$$ (21)

x=1,m=1,n=1.

Using equations (2), (3), and (4),

$$\frac{C_{X}^{A}}{C_{X}^{*B}}=k_{D}+{\left({\mathrm{Kk}}_{D}\right)}^{1/2}{\left(C_{X}^{*B}\right)}^{-1/2}$$ (22)

A plot of $\frac{C_{X}^{A}}{\;C_{X}^{*B}}\;\mathrm{vs}\;\frac{1}{{\left(C_{X}^{*B}\right)}^{\frac{1}{2}}}$ will give a straight line.

When both dimerization and ionization reactions are taking place in solvents A and B respectively, then equation (2), (3), and (4) above are written as ^3^:

$$\frac{C_{X}^{A}}{C_{X}^{*B}}=k_{D}\left(1-\alpha \right)+{{\text{2k}}_{D}}^{2}K{\left(1-\alpha \right)}^{2}{C_{X}}^{*B}$$ (23)

When a solution or mixture is put in a separatory funnel and shaken with an immiscible solvent, the solutes distribute in part, into the two phases. ^26^ At equilibrium, the quantitative relation between the amounts of solute in the two phases is constant, and this is shown by the magnitude, k _D_, called the distribution coefficient/partition coefficient ( LibreTexts). This technique was applied in the separation of morphine in ethyl acetate-water. When equal volumes of organic and aqueous layers are used, the partition coefficient, k _D_, gives the ratio of particles in each layer. The k _D_ of morphine was reported to be 6. This shows that there will be six times as many morphine molecules in the solvent A phase as there are in the solvent B phase. The partition coefficient expresses the miscibility of the compound in the organic and aqueous phases. It is dependent on the solvents system used. Morphine has a k _D_ of 2 in petroleum ether-water, and a k _D_ of 0.33 in diethyl ether-water.

Generally, when the k _D_ is less than 1 (k _D_ <1), the compound distributes into the aqueous phase more than the organic phase. ^23^ When k _D_ = 1, the solute separates equally in the two phases that are involved, and it could be removed completely from the mixture. This applies when the bulk contaminant where the solute is seized is different from the two liquids that are used to form the binary solvent. This condition has been used in the solvent extraction of gold from alkaline cyanide solution by tetradecylmethylbenzylammonium chloride. ^24^ For all cases, when k _D_ > 1, complete separation of the solute from the contaminant can be done in many times.

The Partition coefficient technique has been applied in finding the ‘effective partition coefficient’ (k _D_) for silicon impurities. ^23^ As reported, the process uses the impurities solubility difference in solid and liquid silicon called effective partition coefficient (k _D_). The measured impurities profiles in silicon are compared with the theoretically calculated impurities profiles to determine the effective partition coefficient. In studying the solidification process, the k _D_ technique is applied for the determination of the silicon impurities.

According to Kodolikar and Bhatkhande, ^25^ solvent extraction is an important separation technique in chemical industrial separation and recovery. Thus, selectivity is defined as the ability of a solvent to extract one component of the mixture that contains more than one component, in preference to the other component(s), and partition coefficient shows the measure of the relative selectivity of components for each other. ^25^ Selection of a solvent is considered the main factor in the making of a successful solvent extraction process, as it invariably determines the separation efficiency. The equilibrium data generated relates to selectivity and partition coefficient, for each individual constituent in the system. ^25^ The concept used by Kodolikar and Bhatkhande ^25^ proposes distribution coefficients calculation. Solvent extraction technology is used for the purification and concentration of organic substances, for both energy savings and for environmental protection ( Britannica). The miscibility of oxalic acid with water at 303.15K is low. The miscibility is 12% in mass fraction of anhydrous oxalic acid and gives a dilute solution. This shows that the solvent extraction process is necessary for the removal of oxalic acid from wastewaters. ^26^ The phenomenon of the partition coefficient is a direct consequence of the laws of thermodynamics. The driving force, which is defined in the partition coefficient (k _D_) values, shows the efficiency of the application of this principle in solvent extraction, in beneficiation of minerals. The value of k _D_ depends on the relative solubility of the solute in the two immiscible solvents. When the partition coefficient, k _D_ > 1, total extraction of the solute is made as many times as possible. Also when k _D_< 1, the compound partitions into the aqueous layer more than it distributes into the organic layer ( LibreTexts).

### Application of partition coefficient technique

Solvent extraction is an important method of beneficiation of minerals ( Study). Also log k _D_ (partition coefficient) was used in drug recovery, beneficiation, and development. ^24^ Body cells are likened to mechanical filters which are used to create two partitions like in solvent extraction in the use of organic solvent and aqueous solvent. Partition coefficient describes the relative solubilities of a solute in binary immiscible solvent. In drug development, body cells are likened to mechanical filters which could give the relative sizes of the material used for the development. This process is similar to solvent extraction with two immiscible solvents. Partition coefficient technique is used in research and development.

### Objective of the research

Among the minerals, compounds, and substances nature blessed mankind with are oxalic acid and succinic acid. The continued use and recycling of these compounds to their pure states necessitates that research determines the efficient method for the purification, beneficiation, and analyses of these compounds. Partition coefficient technique gives good separation effect, as it has a high degree of selectivity and fast mass transfer. It also has low energy consumption, large production capacity, fast action, easy continuous operation and ease of automation. ^29^

The results from this research are shown in Tables 1 – 20 and Figures 1 – 18 below.

**Table 1.** Data for the partition coefficient (k _D_) of oxalic acid in carbon tetrachloride-water at 30°C and atmospheric pressure.

Mass of oxalic acid (g)	Concentration in carbon tetrachloride (A) (C _x_^A^) (mole/litre)	Concentration in water (B) (C _x*_^B^) (mole/litre)	$\frac{C_{X}^{A}}{C_{X}^{*B}}$ (k _D_)
0.4	0.0075	0.1475	0.0508
0.6	0.0075	0.2225	0.0337
0.8	0.0063	0.2888	0.0218
1.0	0.0050	0.3650	0.0137

**Table 2.** Data for the partition coefficient of oxalic acid in carbon tetrachloride-water at 30°C and atmospheric pressure for the plot: $\frac{C_{X}^{A}}{C_{X}^{*B}}$ vs 1/(C _x*_^B^) ^1/2^.

$\frac{C_{X}^{A}}{C_{X}^{*B}}$	1/ √ C _x*_^B^ (mole/litre) ^−1/2^
0.0508	2.6035
0.0337	2.1199
0.0218	1.8608
0.0137	1.6551

**Table 3.** Data for the partition coefficient of oxalic acid in carbon tetrachloride-water at 30°C and atmospheric pressure for the plot $\frac{C_{X}^{A}}{C_{X}^{*B}}$ (1− α ) vs C _x*_^B^(1− *α*).

$\frac{C_{X}^{A}}{C_{X}^{*B}}$	$\frac{C_{X}^{A}}{C_{X}^{*B}}$ (1− α )	C _x*_^B^ (mole/litre)	C _x*_^B^(1− α ) (mole/litre)
0.0508	0.0493	0.1475	0.1430
0.0337	0.0327	0.2225	0.2158
0.0218	0.0211	0.2888	0.2800
0.0137	0.0133	0.3650	0.3539

**Table 4.** Data for the partition coefficient (k _D_) for oxalic acid in the binary solvents: diethyl ether-water at 30°C and atmospheric pressure.

Mass of oxalic acid (g)	Concentration in diethyl ether (C _x_^A^) (mole/litre)	Concentration in water (C _x_^*B^) (mole/litre)	$\frac{C_{X}^{A}}{C_{X}^{*B}}$ (k _D_)
0.4	0.0050	0.1400	0.0357
0.6	0.0050	0.1925	0.0260
0.8	0.0125	0.3125	0.0400
1.0	0.0175	0.3350	0.0522

**Table 5.** Data for the partition coefficient of oxalic acid in diethyl ether-water at 30°C and atmospheric pressure, for the plot $\frac{C_{X}^{A}}{C_{X}^{*B}}$ vs 1/(C _x*_^B^) ^1/2^ from equation (22).

$\frac{C_{X}^{A}}{C_{X}^{*B}}$	1/ √ C _x*_^B^ (mole/litre) ^−1/2^
0.0357	2.6724
0.0260	2.2795
0.0400	1.7889
0.0522	1.7277

**Table 6.** Data for the partition coefficient of oxalic acid in diethyl ether-water at 30°C and atmospheric pressure for the plot of equation (23).

$\frac{C_{X}^{A}}{C_{X}^{*B}}$	$\frac{C_{X}^{A}}{C_{X}^{*B}}$ (1− α )	C _x*_^B^ (mole/litre)	C _x*_^B^(1− α ) (mole/litre)
0.0357	0.0356	0.1400	0.1395
0.0260	0.0259	0.1925	0.1918
0.0400	0.0399	0.3125	0.3114
0.0522	0.0520	0.3350	0.3338

**Table 7.** Data for the partition coefficient (k _D_) for oxalic acid in n-hexane-water at 30°C and atmospheric pressure.

Mass of oxalic acid (g)	Concentration in n-hexane (C _x_^A^) (mole/litre)	Concentration in water (C _x_^*B^) (mole/litre)	$\frac{C_{X}^{A}}{C_{X}^{*B}}$ (k _D_)
0.4	0.0075	0.2300	0.0326
0.6	0.0025	0.1650	0.0152
0.8	0.0050	0.2975	0.0168
1.0	0.0050	0.3675	0.0136

**Table 8.** Data for the partition coefficient of oxalic acid in n-hexane-water at 30°C and atmospheric pressure for the plot $\frac{C_{X}^{A}}{C_{X}^{*B}}$ vs 1/(C _x*_^B^) ^1/2^, equation of 22.

$\frac{C_{X}^{A}}{C_{X}^{*B}}$	1/ √ C _x*_^B^ (mole/litre) ^−1/2^
0.0326	2.0851
0.0152	2.4618
0.0168	1.8335
0.0136	1.6496

**Table 9.** Data for the partition coefficient of oxalic acid in n-hexane-water at 30°C and atmospheric pressure for the plot: $\frac{C_{X}^{A}}{C_{X}^{*B}}$ (1− α ) vs C _x*_^B^(1− α ), equation (23).

$\frac{C_{X}^{A}}{C_{X}^{*B}}$	$\frac{C_{X}^{A}}{C_{X}^{*B}}$ (1− α )	C _x*_^B^ (mole/litre)	C _x*_^B^(1− α ) (mole/litre)
0.0326	0.0325	0.2300	0.2296
0.0152	0.0152	0.1650	0.1647
0.0168	0.0168	0.2975	0.2969
0.0136	0.0136	0.3675	0.3668

**Table 10.** The partition coefficient, k _D_, dimerization constant, K, and ionization constant, α , for oxalic acid in the binary solvents, carbon tetrachloride-water, diethyl ether-water and n-hexane-water respectively at 30°C and atmospheric pressure.

Oxalic acid	Partition coefficient, k _D_	Dimerization constant, K	Ionization constant, α	Association and ionization
Carbon tetrachloride-water	0.07383	−15.7092	0.0303	−16.6974
Diethyl ether-water	0.0173	144.0167	0.0035	144.98
n-hexane-water	0.02793	20.2798	0.0019	−20.296

**Table 11.** Data for the partition coefficient (k _D_) for succinic acid in the binary solvent of carbon tetrachloride-water at 30°C and atmospheric pressure.

Mass of succinic acid (g)	Concentration in carbon tetrachloride (C _x_^A^) (mole/litre)	Concentration in water (C _x*_^B^) (mole/litre)	$\frac{C_{X}^{A}}{C_{X}^{*B}}$ (k _D_)
0.4	0.0075	0.1775	0.0423
0.6	0.0025	0.2950	0.0085
0.8	0.0025	0.4550	0.0055
1.0	0.0050	0..4050	0.0123

**Table 12.** Data for the partition coefficient of succinic acid in carbon tetrachloride-water at 30°C and atmospheric pressure for the plot $\frac{C_{X}^{A}}{C_{X}^{*B}}$ vs 1/(C _x*_^B^) ^1/2^ equation (22).

$\frac{C_{X}^{A}}{C_{X}^{*B}}$	1/ √ C _x*_^B^ (mole/litre) ^−1/2^
0.0423	2.3736
0.0085	1.8413
0.0055	1.4826
0.0123	1.5713

**Table 13.** Data for the partition coefficient of succinic acid in carbon tetrachloride-water at 30°C and atmospheric pressure for the plot k _D_(1− α ) vs C _x*_^B^(1− α ).

$\frac{C_{X}^{A}}{C_{X}^{*B}}$	$\frac{C_{X}^{A}}{C_{X}^{*B}}$ (1− α )	C _x*_^B^ (mole/litre)	C _x*_^B^(1− α ) (mole/litre)
0.0423	0.0411	0.1775	0.1725
0.0085	0.0083	0.2950	0.2866
0.0055	0.0053	0.4550	0.4421
0.0123	0.0120	0.4050	0.3935

**Table 14.** Data for the partition coefficient (k _D_) for succinic acid in the binary solvent: diethyl ether-water at 30°C and atmospheric pressure.

Mass of succinic acid (g)	Concentration in diethyl ether (C _x_^A^) (mole/litre)	Concentration in water (C _X*_^B^) (mole/litre)	$\frac{C_{X}^{A}}{C_{X}^{*B}}$ (k _D_)
0.4	0.0075	0.1675	0.0448
0.6	0.0025	0.1800	0.0139
0.8	0.0095	0.3100	0.0306
1.0	0.0050	0.3950	0.0127

**Table 15.** Data for the partition coefficient of succinic acid in diethyl ether-water at 30°C and atmospheric pressure, for the plot $\frac{C_{X}^{A}}{C_{X}^{*B}}$ vs 1/(C _x*_^B^) ^1/2^ for equation (22).

$\frac{C_{X}^{A}}{C_{X}^{*B}}$	1/ √ C _x*_^B^ (mole) ^−1/2^
0.0448	2.4432
0.0139	2.3568
0.0306	1.7960
0.0127	1.5911

**Table 16.** Data for the partition coefficient of succinic acid in diethyl ether-water at 30°C and atmospheric pressure for the plot $\frac{C_{X}^{A}}{C_{X}^{*B}}$ (1− α ) vs C _x*_^B^(1− α ).

$\frac{C_{X}^{A}}{C_{X}^{*B}}$	$\frac{C_{X}^{A}}{C_{X}^{*B}}$ (1− α )	C _x*_^B^ (mole/litre)	C _x*_^B^(1− α ) (mole/litre)
0.0448	0.0433	0.1675	0.1619
0.0139	0.0134	0.1800	0.1740
0.0306	0.0296	0.3100	0.2997
0.0127	0.0123	0.3950	0.3819

**Table 17.** Data for the partition coefficient (k _D_) for succinic acid in the binary solvent: n-hexane-water at 30°C and atmospheric pressure.

Mass of succinic acid (g)	Concentration in n-hexane (C _x_^A^) (mole/litre)	Concentration in water (C _x_^*B^) (mole/litre)	$\frac{C_{X}^{A}}{C_{X}^{*B}}$ (k _D_)
0.4	0.0050	0.1900	0.0263
0.6	0.0025	0.2500	0.0100
0.8	0.0050	0.3175	0.0157
1.0	0.0325	0.4000	0.0813

**Table 18.** Data for the partition coefficient of succinic acid in n-hexane-water at 30°C and atmospheric pressure, for the plot $\frac{C_{X}^{A}}{C_{X}^{*B}}$ vs 1/(C _x*_^B^) ^1/2^ for equation (22).

$\frac{C_{X}^{A}}{C_{X}^{*B}}$	1/ √ C _x*_^B^ (mole/litre) ^−1/2^
0.0263	2.2941
0.0100	2.0000
0.0157	1.7746
0.0813	1.5810

**Table 19.** Data for the partition coefficient of succinic acid in n-hexane-water at 30°C and atmospheric pressure, for the plot $\frac{C_{X}^{A}}{C_{X}^{*B}}$ (1− α ) vs C _x*_^B^(1− α ).

$\frac{C_{X}^{A}}{C_{X}^{*B}}$	$\frac{C_{X}^{A}}{C_{X}^{*B}}$ (1− α )	C _x*_^B^ (mole/litre)	C _x*_^B^(1− α ) (mole/litre)
0.0263	0.0256	0.1900	0.1850
0.0100	0.0097	0.2500	0.2434
0.0157	0.0153	0.3175	0.3091
0.0813	0.0791	0.4000	0.3894

**Table 20.** Values for partition coefficient, k _D_, dimerization constant, K, and ionization constant, α, for succinic acid in the binary immiscible solvents carbon tetrachloride-water, diethyl ether-water and n-hexane-water respectively at 30°C and atmospheric pressure.

Succinic acid	Partition coefficient k _D_	Dimerization constant, K	Ionization constant, α	Association and ionization constant
Carbon tetrachloride-water	−0.0562	−18.5655	0.0284	−19.6457
Diethyl ether-water	0.0427	−18.1611	0.0332	−19.17
n-hexane-water	−0.0427	71.9491	0.0265	75.89

**Figure 1.**
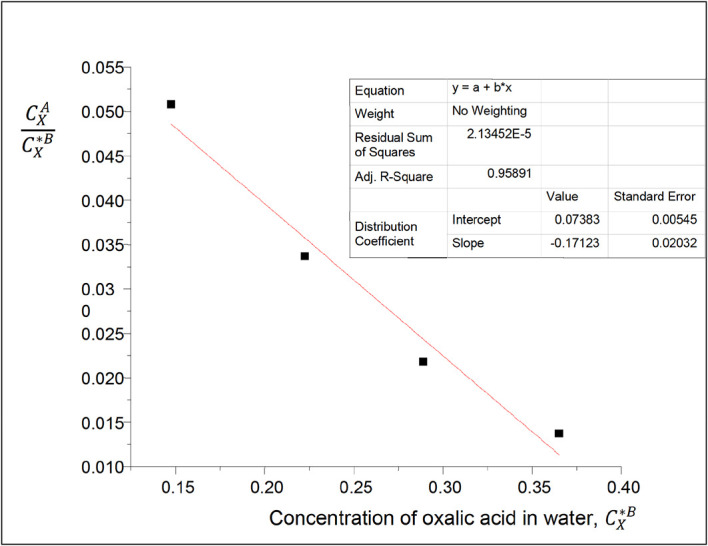
Plot for $\frac{C_{X}^{A}}{C_{X}^{*B}}$ vs C _x*_^B^ at 30°C and atmospheric pressure for oxalic acid in carbon tetrachloride-water, from equation (19).

**Figure 2.**
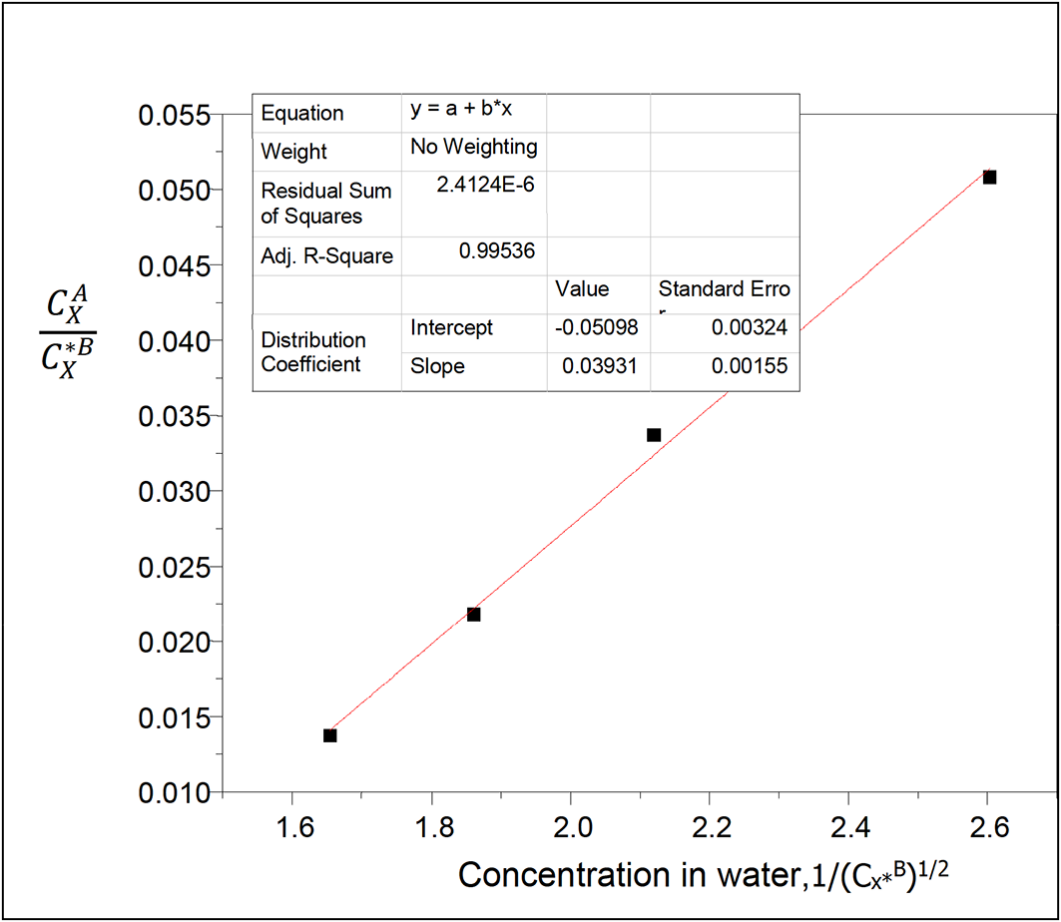
Plot of $\frac{C_{X}^{A}}{C_{X}^{*B}}$ vs 1/(C _x*_^B^) ^1/2^ for the ionization of oxalic acid in water of carbon tetrachloride-water at 30°C and atmospheric pressure from equation (22).

**Figure 3.**
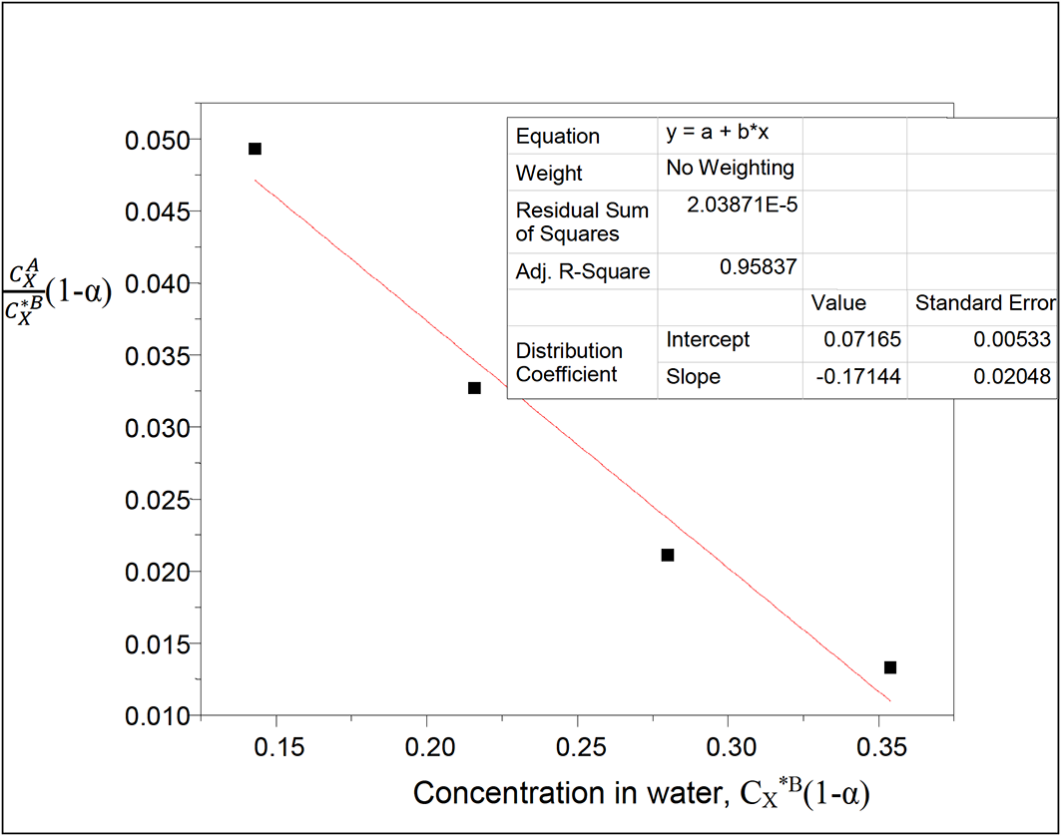
Plot for $\frac{C_{X}^{A}}{C_{X}^{*B}}$ (1− α ) vs C _x*_^B^(1−α) for the association and ionization of oxalic acid in carbon tetrachloride-water at 30°C and atmospheric pressure

**Figure 4.**
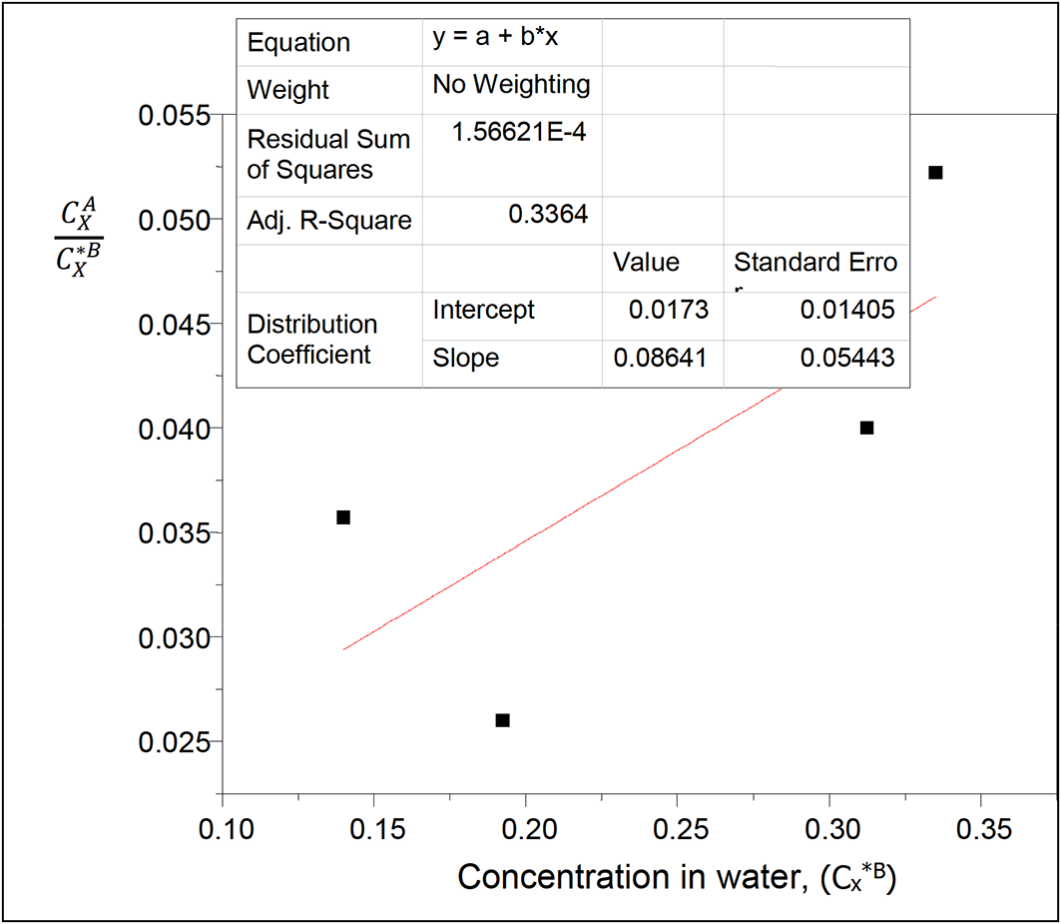
Plot for $\frac{C_{X}^{A}}{C_{X}^{*B}}$ vs C _x*_^B^ for oxalic acid in diethyl ether-water at 30°C and atmospheric pressure, from equation (19).

**Figure 5.**
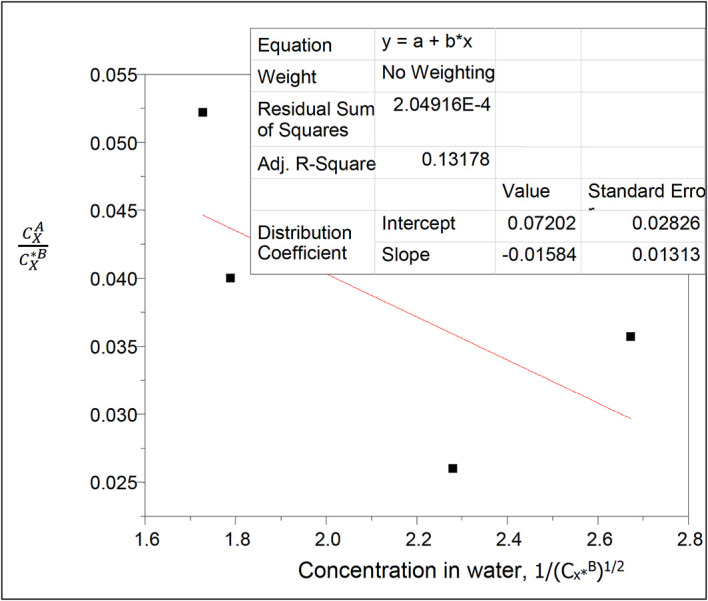
Plot for $\frac{C_{X}^{A}}{C_{X}^{*B}}$ vs 1/(C _x*_^B^) ^1/2^ for the ionization of oxalic acid in water of diethyl ether-water at 30°C and atmospheric pressure, from equation (22).

**Figure 6.**
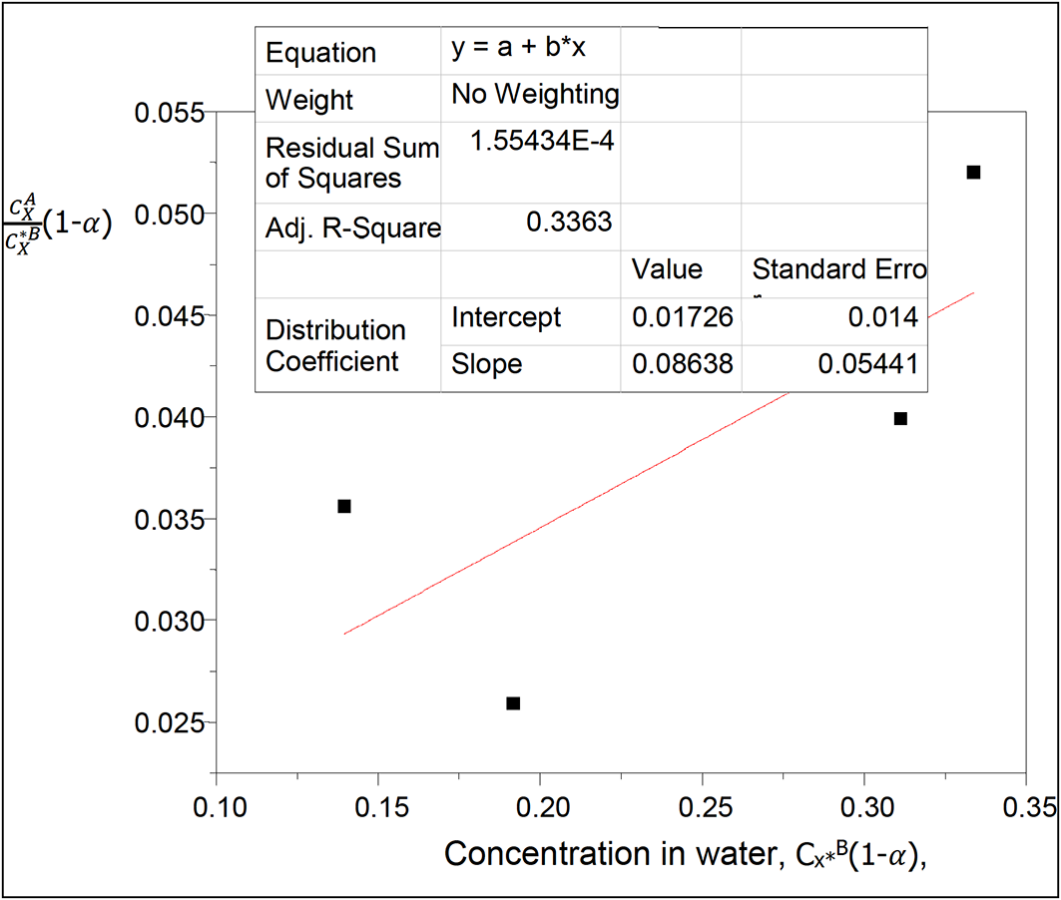
Plot for oxalic acid in diethyl ether-water at 30°C and atmospheric pressure for the plot $\frac{C_{X}^{A}}{C_{X}^{*B}}$ (1−α) vs C _x*_^B^(1−α), equation (23).

**Figure 7.**
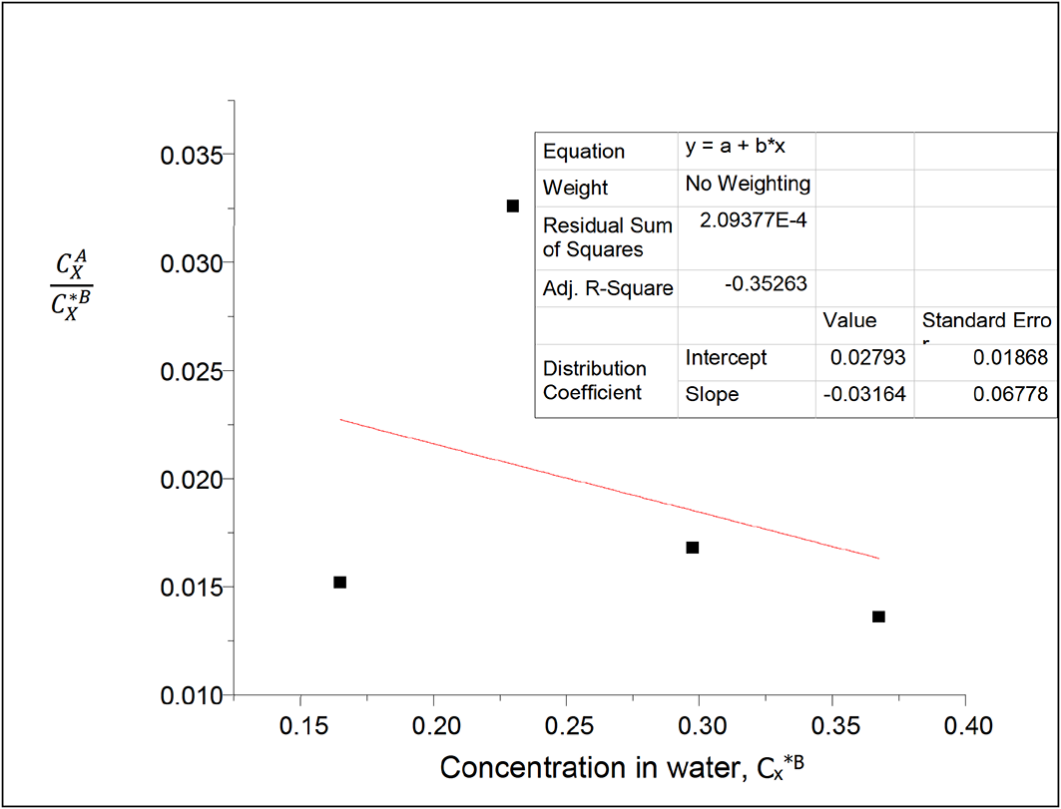
Plot of $\frac{C_{X}^{A}}{C_{X}^{*B}}$ against C _x_^*B^ for oxalic acid in n-hexane-water at 30°C and atmospheric pressure.

**Figure 8.**
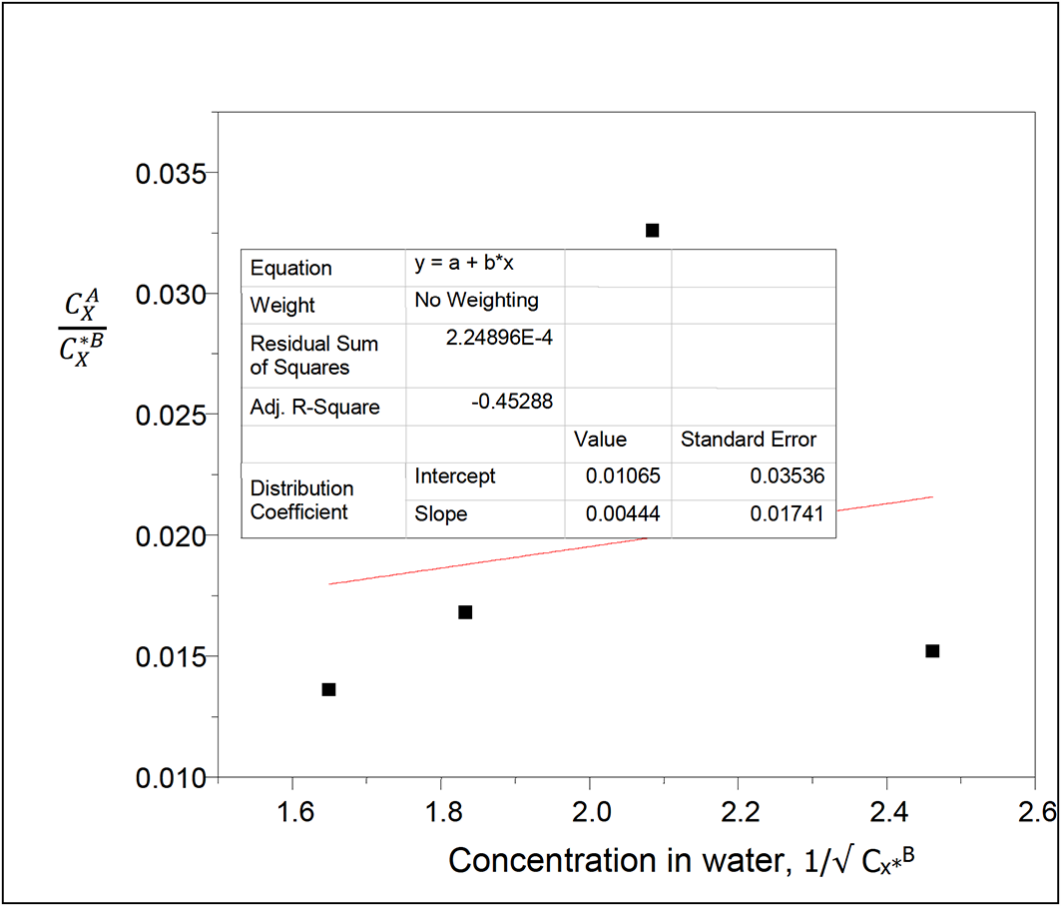
Plot for $\frac{C_{X}^{A}}{C_{X}^{*B}}$ vs 1/(C _x*_^B^) ^1/2^ for oxalic acid in n-hexane-water at 30°C and atmospheric pressure.

**Figure 9.**
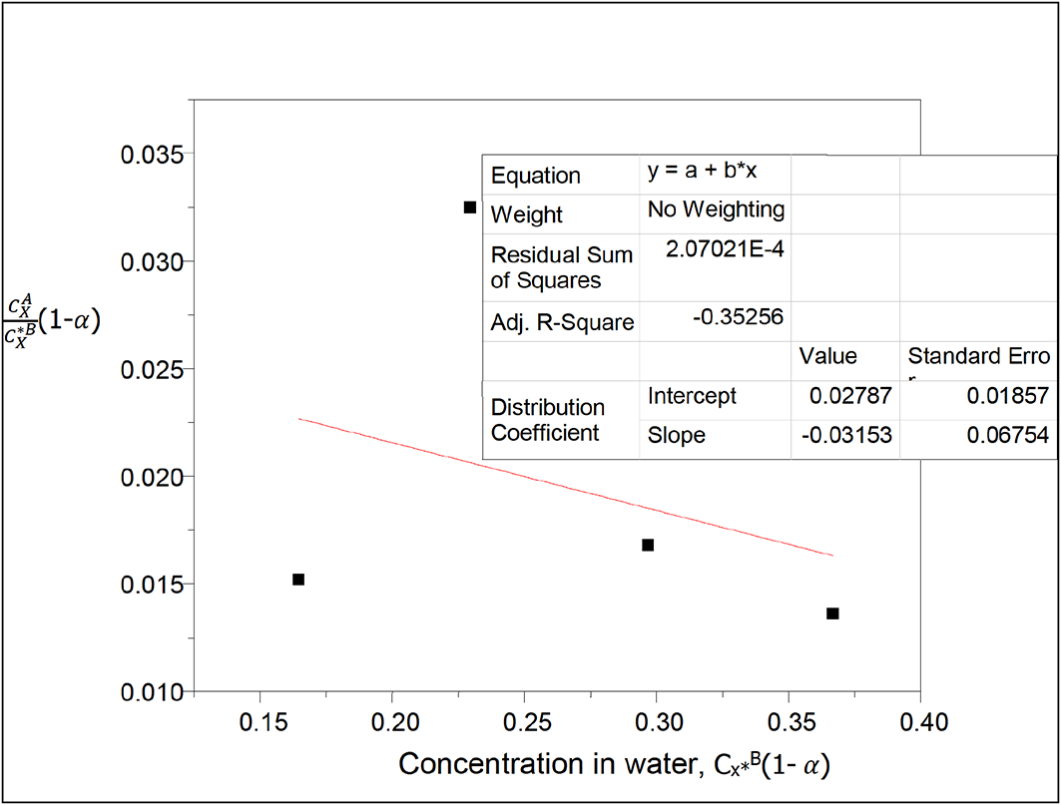
Plot for $\frac{C_{X}^{A}}{C_{X}^{*B}}$ (1− *α*) vs C _x*_^B^(1−α) for oxalic acid in n-hexane-water at 30°C and atmospheric pressure.

**Figure 10.**
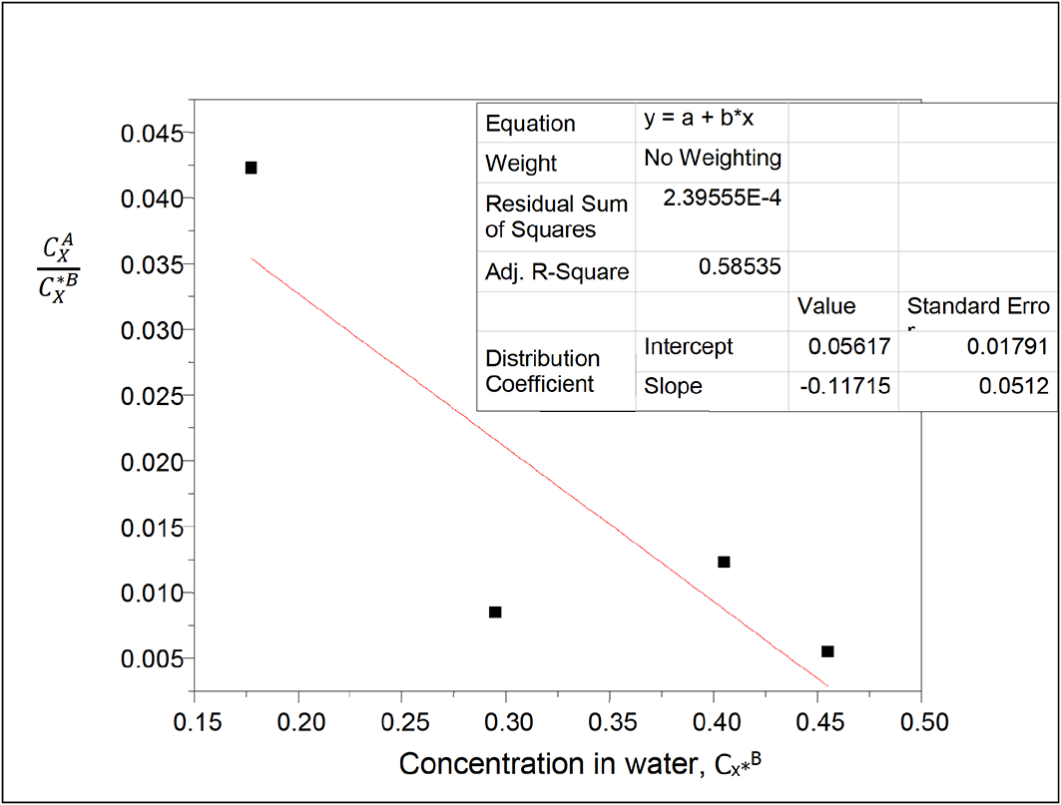
Plot of $\frac{C_{X}^{A}}{C_{X}^{*B}}$ vs C _x*_^B^ for succinic acid in carbon tetrachloride-water at 30°C and atmospheric pressure for equation (19).

**Figure 11.**
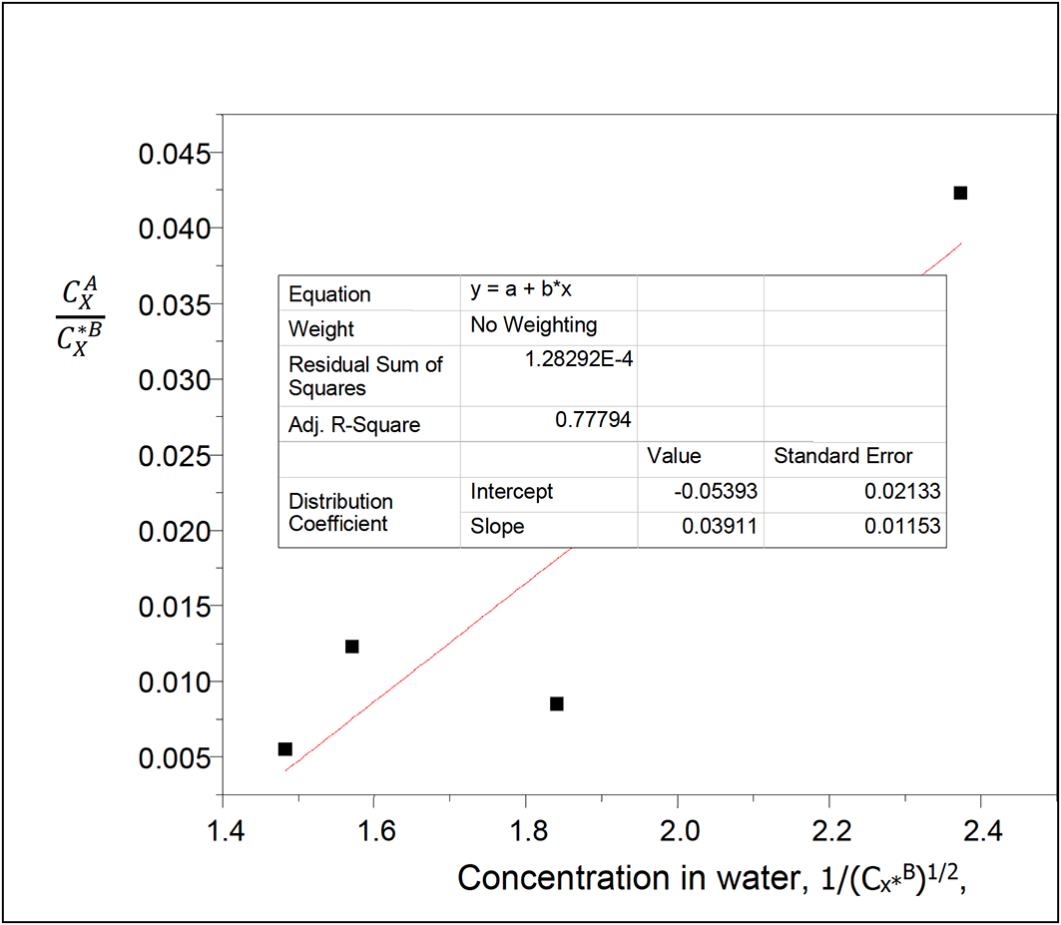
Plot of $\frac{C_{X}^{A}}{C_{X}^{*B}}$ vs 1/(C _x*_^B^) ^1/2^ for the ionization of succinic acid in water of carbon tetrachloride-water at 30°C and atmospheric pressure for equation (22).

**Figure 12.**
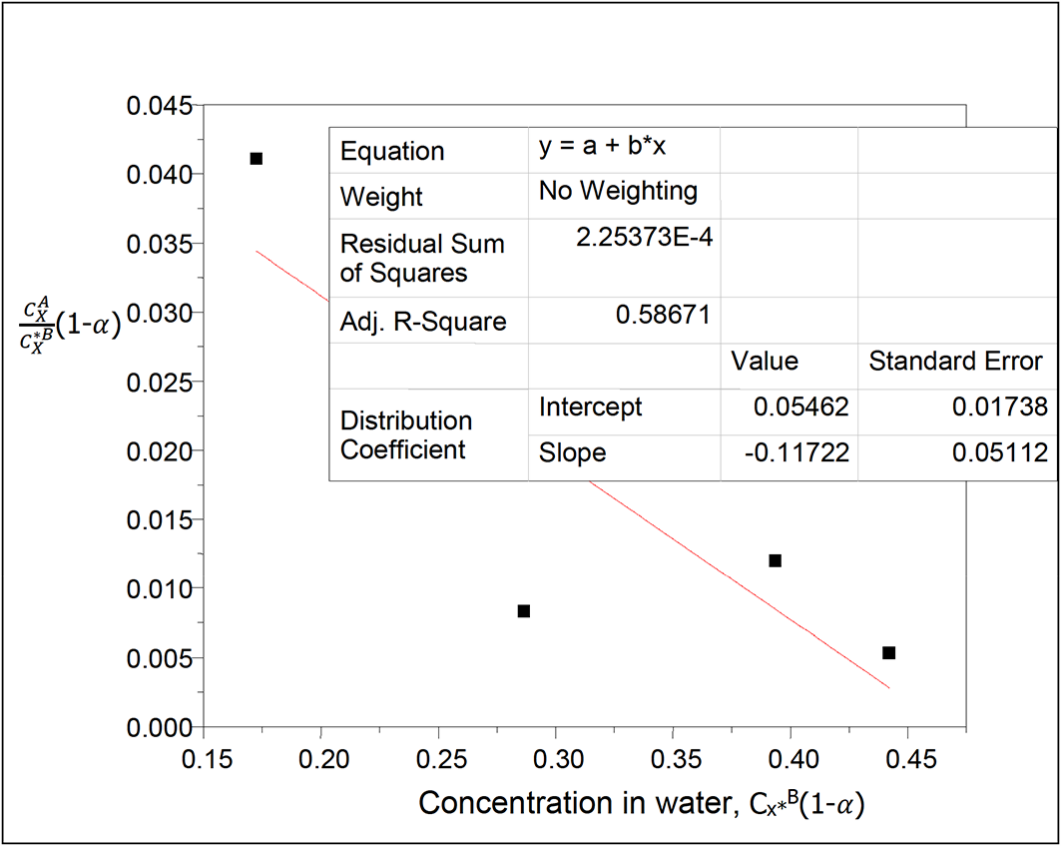
Plot for $\frac{C_{X}^{A}}{C_{X}^{*B}}$ (1− *α*) vs C _x*_^B^(1−α) for the association and ionization of succinic acid in both carbon tetrachloride and water of carbon tetrachloride-water system.

**Figure 13.**
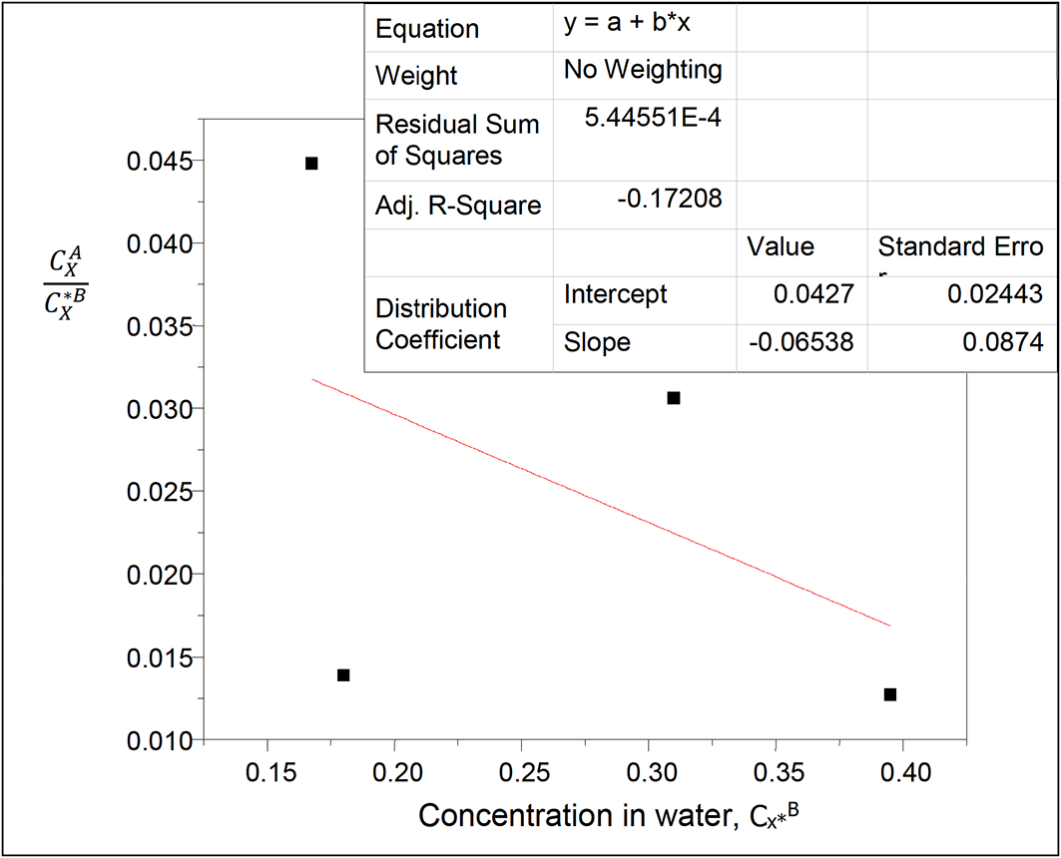
Plot for $\frac{C_{X}^{A}}{C_{X}^{*B}}$ vs C _x*_^B^ for succinic acid in diethyl ether-water at 30°C and atmospheric pressure.

**Figure 14.**
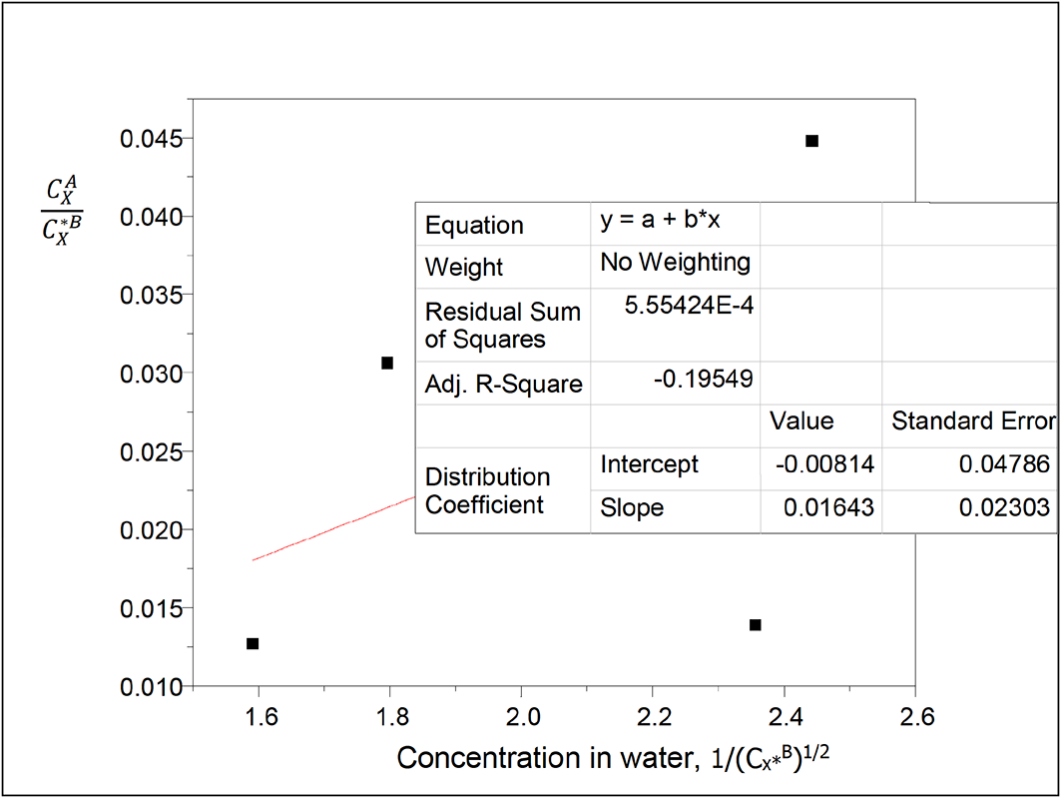
Plot for the partition coefficient of succinic acid in diethyl ether-water at 30°C and atmospheric pressure, for the plot $\frac{C_{X}^{A}}{C_{X}^{*B}}$ vs 1/(C _x*_^B^) ^1/2^, from equation (22).

**Figure 15.**
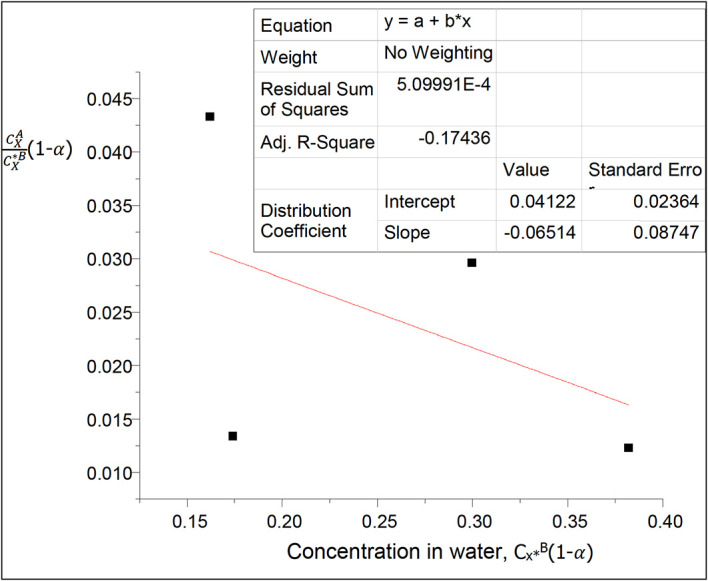
Plot for the partition coefficient of succinic acid in diethyl ether-water at 30°C and atmospheric pressure, for the plot $\frac{C_{X}^{A}}{C_{X}^{*B}}$ (1−α) vs C _x*_^B^(1−α) from equation (23).

**Figure 16.**
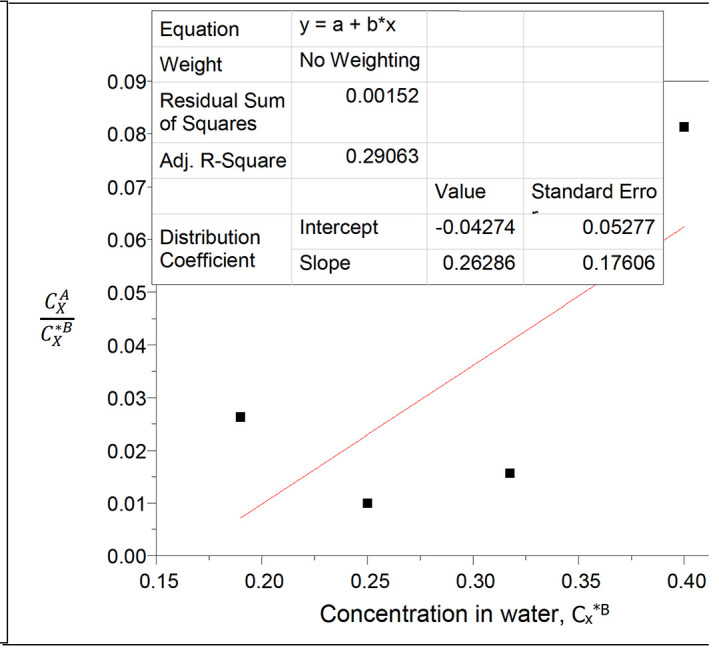
Plot $\frac{C_{X}^{A}}{C_{X}^{*B}}$ vs C _x_^*B^ for succinic acid in n-hexane-water at 30°C and atmospheric pressure.

**Figure 17.**
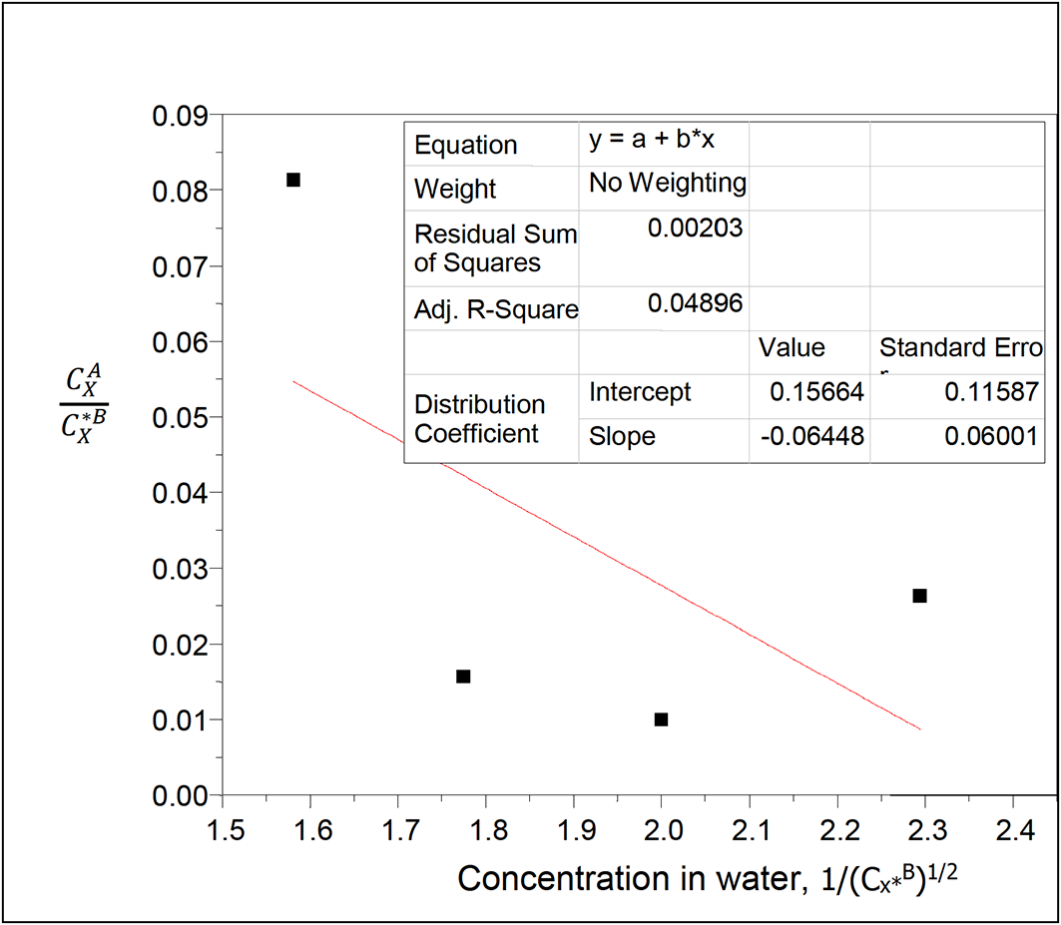
Plot for $\frac{C_{X}^{A}}{C_{X}^{*B}}$ vs 1/(C _x*_^B^) ^1/2^ for succinic acid ionization in water of n-hexane-water at 30°C and atmospheric pressure using equation (22).

**Figure 18.**
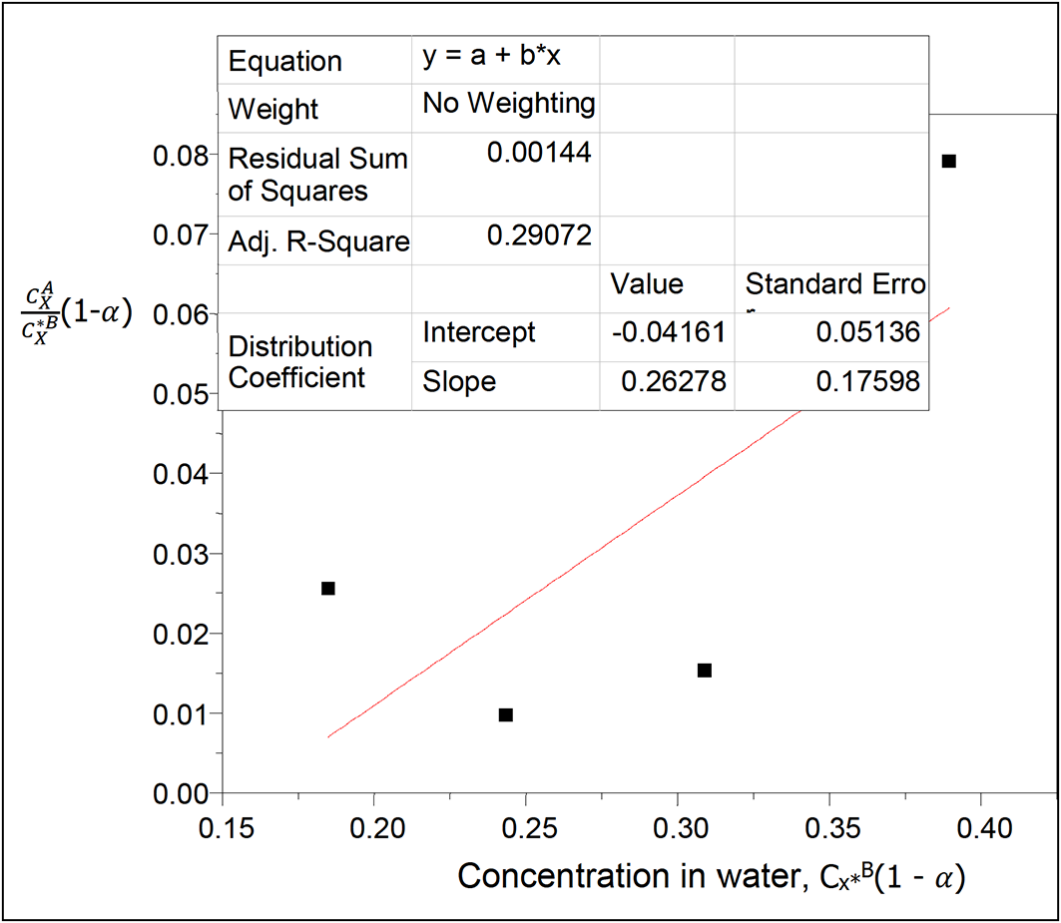
Plot for $\frac{C_{X}^{A}}{C_{X}^{*B}}$ (1 − *α*) vs C _x*_^B^(1 − α) for succinic acid in n-hexane-water at 30°C and atmospheric pressure.

## Methods

### Brief note

The materials for the experiment are AnalaR Grade reagents ^27^ of oxalic acid, succinic acid, carbon tetrachloride, diethyl ether, n-hexane, phenolphthalein, 0.5 M sodium hydroxide, and distilled water. A Drawell digital precision balance, model FA1204, and origin 50 software were used for the measurement of reagents and the creation of graphs, respectively. The various solutions of the binary immiscible solvents were prepared to get the stock solutions as described below. ^3^^,^^26^ This method was used in the analyses of acetic and succinic acids in binary immiscible solvents, as described by Veronica *et al*. ^1^ All measurements were done at 30°C and atmospheric pressure.

### Preparation of sodium hydroxide solution (0.5M NaOH)

20 g of sodium hydroxide solid was weighed into 500ml of distilled water in a conical flask. The mixture was shaken well by hand for complete dissolution of the solute. The mixture was then made up to 1000 ml (1litre) mark with more distilled water, in the conical flask.

### Preparation of phenolphthalein solution

0.5 g of phenolphthalein solid was measured into 50 ml of 90% ethanol. The mixture was shaken vigorously by hand until the phenolphthalein dissolved completely. 50 ml of distilled water was then poured into the solution to ensure proper dissolution of the mixture. This solution gives the phenolphthalein solution (the indicator) for titrations.

### Preparation of binary immiscible solvents I and the determination of partition coefficient, k _D_ from the binary immiscible solvent, method I

25.00 ml of distilled water was measured using a clean measuring cylinder into a separating funnel. 25.00 ml of carbon tetrachloride was measured using another clean measuring cylinder, and was poured into the same separating funnel with the water. The mixture was shaken well. This mixture forms the binary immiscible solvent.

0.40 g of oxalic acid was measured out using a weighing balance. It was weighed into a binary immiscible solvent system of 25.00 ml of water and 25.00 ml of carbon tetrachloride in a separating funnel. The separating funnel was shaken up for 15-20 minutes by hand, for proper mixing of the content. The system was then allowed to stand for 60 minutes, until equilibrium was reached and separation of the organic and aqueous layer achieved. The lower organic layer was separated by hand, by pouring it gradually into a clean beaker while the upper aqueous layer was separated (decanted) into another clean beaker. These steps were repeated for 0.60 g, 0.80 g, and 1.0 g of oxalic acid to create four systems.

10.00 ml of the organic phase of the 0.4 g system was titrated with 0.5 mol sodium hydroxide per 1 litre of water, with phenolphthalein as the indicator. The titration was carried out until a pink colour was obtained as the end point was reached. The volume of sodium hydroxide used was recorded. 10.00 ml of the aqueous phase was also titrated with 0.5 mol sodium hydroxide per 1 litre of water, again using phenolphthalein as an indicator until the end point was reached. Again, the volume of sodium hydroxide used was recorded. The experiment was repeated separately for the 0.60 g, 0.80 g, and 1.0g systems of oxalic acid. The concentrations of oxalic acid in the two phases were calculated.

### Preparation of binary immiscible solvents II and the determination of partition coefficient, k _D_ from the binary immiscible solvent

The method for preparation of immiscible solvents I (method I) was exactly repeated using diethyl ether and water instead of carbon tetrachloride and water, with 0.40 g, 0.60 g, 0.80 g, and 1.0 g of oxalic acid respectively. The concentrations of oxalic acid in the two layers were also calculated.

### Preparation of binary immiscible solvents III and the determination of partition coefficient, k _D_ from the binary immiscible solvent

The method for preparation of immiscibly solvents I (method I) was exactly repeated using n-hexane and water instead of carbon tetrachloride and water, with 0.40 g, 0.60 g, 0.80 g, and 1.0 g of oxalic acid respectively. The concentrations of oxalic acid in the two layers were also calculated.

### Preparation of binary immiscible solvents IV and the determination of partition coefficient, k _D_ from the binary immiscible solvent (method II)

25.00 ml of distilled water was measured using a clean measuring cylinder, and poured into a separating funnel. 25.00 ml of carbon tetrachloride was measured using another clean measuring cylinder and poured into the same separating funnel above. The mixture in the separating funnel was shaken well. This mixture forms the binary immiscible solvent.

0.40 g of succinic acid was measured out using the weighing balance. It was introduced into binary immiscible solvent system of 25.00 ml of water and 25.00 ml of carbon tetrachloride in the separatory funnel. The system was shaken vigorously for 15-20 minutes by hand. It was then allowed to stand for 60 minutes until equilibrium was reached and the separation of the organic and aqueous layers was achieved. The lower organic layer was decanted into a clean beaker while the upper aqueous layer was discharged into another clean beaker. All the activities here were done manually. These steps were repeated for 0.60 g, 0.80 g, and 1.0 g of succinic acid to create four systems.

10.00 ml of the organic phase of the 0.4 g system was titrated with 0.5 mol sodium hydroxide per 1 litre of water, with phenolphthalein as the indicator. The titration was carried out until a pink colour was obtained as the end point was reached. The volume of sodium hydroxide used was recorded. 10.00 ml of the aqueous phase was also titrated with 0.5 mol sodium hydroxide per 1 litre of water, again using phenolphthalein as an indicator until the end point was reached. Again, the volume of sodium hydroxide used was recorded. The experiment was repeated separately for the 0.60 g, 0.80 g, and 1.0 g systems of succinic acid. The concentrations of succinic acid in the two phases were calculated.

### Preparation of binary immiscible solvents V and the determination of partition coefficient, k _D_ from the binary immiscible solvent, method II

Method II above was exactly repeated using diethyl ether and water instead of carbon tetrachloride and water, with 0.40 g, 0.60 g, 0.80 g, and 1.0 g of succinic acid respectively. The concentrations of oxalic acid in the two layers were also calculated.

### Preparation of binary immiscible solvents VI and the determination of partition coefficient, k _D_ from the binary immiscible solvent, method II

Method II was also repeated using n-hexane and water instead of carbon tetrachloride and water, with 0.40 g, 0.60 g, 0.80 g, and 1.0 g of succinic acid respectively. The concentrations of oxalic acid in the two layers were also calculated.

### Summary on the determination of partition coefficient

Methods I and II were used to determine the partition coefficients of the acids in the binary immiscible solvents. Every system of each binary solvent has two phases, aqueous and organic, which were separated manually by pouring each partition gradually into a beaker. The partition coefficient is given by “concentration of the acid in the organic layer (layer A) / concentration of the acid in the aqueous layer (layer B)”. Taking the case of oxalic acid in n-hexane-water system, the partition coefficient is given as, “concentration of oxalic acid in n-hexane/concentration of oxalic acid in water”.

The equation:

$$C_{A}V_{A}/C_{B}V_{B}=\text{Mole ratio}$$

was used to calculate the concentration or the relative amount of the acid in the two solvents of the binary immiscible solvent systems.

The reaction of oxalic acid with NaOH gives:

$$\text{HOOCCOOH}+\text{2NaOH}\to \text{NaOOCCOONa}+{\text{2H}}_{2}O$$

where the volume of base (NaOH) used for the titration = V _B_

the concentration of the base, C _B_ = 0.5 M

The volume of the acid (oxalic acid) = V _A_ = 10 ml

The concentration of acid, C _A_, was calculated.

## Results and discussion

The values of the partition coefficient (k _D_) for oxalic acid in carbon tetrachloride-water at 30°C and atmospheric pressure were calculated as shown in Table 1.

Table 1 shows the dissolution of oxalic acid in carbon tetrachloride and in water, and the degree of its subsequent extraction. The partition coefficient values show that the complete extraction of oxalic acid in this system could be done in many steps, and that oxalic acid dissolves more in water than in carbon tetrachloride. As the concentration of oxalic acid increases in water, the partition coefficient value decreases. In a situation where the contaminant is a material that is different from both water and carbon tetrachloride, the carbon tetrachloride-water system is also used to extract the oxalic acid from such contaminant.

Intercept, k _D_ = 0.07383

slope = −0.17123, = 2k _D_^2^K, K = −15.7092

K is the constant for the formation of oxalic acid dimers in carbon tetrachloride.

The magnitude of K is greater than that of k _D_. Also, the higher the magnitude of K, the more the formation of oxalic acid dimers.

Oxalic acid ionizes in water to form the oxalate, as shown in equations (9) and (10).

This table shows how the partition coefficient values relate with the concentration of oxalic acid in water. As the concentration of oxalic acid decreases, the partition coefficient value decreases too.

From Figure 2, k _D_ = −0.05098

slope = 0.03931 = (Kk _D_) ^1/2^

K = 0.0303 = α

α is the reaction equilibrium constant represented in the ionization process. It gives the degree of the ionization of oxalic acid in water in the carbon tetrachloride-water system.

Both dimerization and ionization reactions are considered with oxalic acid in the two liquids, carbon tetrachloride and water respectively.

Equation (23) is used.

A plot of k _D_(1− α ) vs C _X_^*B^(1− α ) will give a straight line, α is the degree of ionization of oxalic acid in water

Thus, 1− α= 0.9697

This table presents the effect of ionization on the partition coefficient of oxalic acid in carbon tetrachloride-water. It also shows the effect of ionization on the concentration of oxalic acid in water. The concentration of oxalic acid in water increases linearly, while the partition coefficient value decreases in a similar manner.

k _D_ = 0.07165

slope = −0.17144 = 2k _D_^2^K = 0.010267445K

K = −16.6974

K is the combined reaction equilibrium constant which is represented here in dimerization and ionization processes of oxalic acid in carbon tetrachloride-water system.

The values of the partition coefficient (k _D_) for oxalic acid in diethyl ether-water at 30°C and atmospheric pressure were calculated as shown in Table 4.

Table 4 shows the dissolution of oxalic acid in diethyl ether and in water. The partition coefficient values show a gradual increase as the concentration of oxalic acid in the system increases. Oxalic acid dissolves more in water than in diethyl ether.

Intercept, k _D_ = 0.0173

slope = 2k _D_^2^K = 0.08641

K = 144.0167

K is the reaction equilibrium constant represented with the formation of oxalic acid dimers in diethyl ether. It gives the degree of dimerization of oxalic acid in diethyl ether.

This table presents the partition coefficient values of oxalic acid in diethyl ether-water and the concentration of oxalic acid in water. As the concentration of oxalic acid decreases in water, the partition coefficient value increases.

Intercept, k _D_ = 0.07202

slope = −0.01584 = (Kk _D_) ^1/2^

K = α = 0.0035

This reaction equilibrium constant K represented by α , gives the degree of ionization of oxalic acid in water in this system.

Equation (23) is used.

A plot of $C_{X}^{A}$ / $C_{X^{*}}^{B}$ (1− α ) vs $C_{X}^{*B}$ (1− α ) is a straight line, α is the degree of ionization of oxalic acid in water.

Both the ionization of oxalic acid in water and its dimerization in diethyl ether respectively are considered.

Thus, 1− α= 0.9965

Table 6 shows the effect of ionization on the partition coefficient of oxalic acid in diethyl ether-water. The concentration of oxalic acid increases as the partition coefficient values increase.

k _D_ = 0.01726

slope = 0.08638 = 2k _D_^2^K = 0.0005958152K

K = 144.98

This value gives the combined reactions constant for oxalic acid in diethyl ether-water.

The values of the partition coefficient (k _D_) for oxalic acid in n-hexane-water at 30°C and atmospheric pressure were calculated as shown in Table 7.

Table 7 shows the dissolution of oxalic acid in n-hexane and in water in the n-hexane-water system. The partition coefficient value of oxalic acid in this system decreases with the increase in concentration of oxalic acid in the system. Additionally, oxalic acid dissolves more in water than in n-hexane.

Intercept, k _D_ = 0.02793

slope = 2k _D_^2^K = −0.03164

K = 20.2798

K is the reaction constant for the degree of formation of the oxalic acid dimers in n-hexane.

This table relays the concentration of oxalic acid in water and the partition coefficient values of oxalic acid in n-hexane-water. The concentration of oxalic acid in water decreases as the partition coefficient values decrease.

Intercept, k _D_ = 0.01065

slope = 0.00444 = (Kk _D_) ^1/2^

K = α = 0.0019

This reaction equilibrium constant gives the extent of ionization of oxalic acid molecules in water in this system.

Equation (23) is used.

A plot of $\frac{C_{X}^{A}}{C_{X}^{*B}}$ (1− α ) vs $C_{X}^{*B}$ (1− α ) is a straight line, α is the degree of ionization of oxalic acid in water.

Both the ionization of oxalic acid in water, and its dimerization in n-hexane are considered.

Thus, 1 − α= 0.9981

Table 9 shows the effect of ionization on the partition coefficient values and on the concentration of oxalic acid in water in the n-hexane-water system. The concentration of oxalic acid in water increases as the partition coefficient value decreases.

k _D_ = 0.02787

slope = −0.03153 = 2k _D_^2^K = 0.0015534738K

K = −20.296

This equilibrium constant gives the combined equilibrium constant for both dimerization and ionization of oxalic acid in n-hexane and water respectively in this system.

From Table 10, oxalic acid has the highest partition coefficient value of 0.07383 in carbon tetrachloride-water and the lowest dimerization constant magnitude of 15.7092 also in carbon tetrachloride-water. This makes carbon tetrachloride-water the best binary solvent for the analysis of oxalic acid. Dimerization reactions occurred in the three systems with the highest dimerization constant magnitude of 144.0167 in the diethyl ether-water system. There were low ionization reactions in the three systems. The interfering reactions of dimerization and ionization affect the molecular distributions of oxalic acid in the binary solvents.

The values of the partition coefficient (k _D_) for succinic acid in carbon tetrachloride-water at 30°C and atmospheric pressure were calculated as shown in Table 11.

A plot of $\frac{C_{X}^{A}}{C_{X}^{*B}}$ against $C_{X}^{*B}$ from equation (19) gave a straight line ( Figure 10) with intercept k _D_ and slope equal to 2k _D_^2^K. The dimerization constant, K, was calculated using the slope of the plot according to the relations:

$$K=\text{Slope}/{{\text{2k}}_{D}}^{2}$$

Table 11 shows the dissolution of succinic acid in carbon tetrachloride and in water in this system. Succinic acid dissolves more in water than in carbon tetrachloride. Additionally, the partition coefficient value decreases as the concentration of succinic acid in the system increases.

k _D_ = 0.05617

slope = −0.11715

K = −18.5655

K is the equilibrium constant for the formation of succinic anhydride in carbon tetrachloride.

Table 12 relates the concentration of succinic acid in water and the partition coefficient values in carbon tetrachloride-water. The concentration of succinic acid in water decreases as the partition coefficient value decreases.

k _D_ = −0.05393

slope = 0.03911 = (Kk _D_) ^1/2^

K = 0.0284 = α

The equilibrium constant K represented by the ionization constant α gives the degree of ionization of succinic acid in water of carbon tetrachloride-water system.

Both dimerization and ionization reactions occurred with succinic acid solute in the two solvents respectively.

Equation (23) is used.

A plot of $\frac{C_{X}^{A}}{C_{X}^{*B}}$ (1− α ) vs C _X_^*B^(1− α ) gives a straight line, α is the degree of ionization of the succinic acid in water.

Thus, (1− α ) = 0.9716.

Table 13 shows the effect of ionization on the partition coefficient values and on the concentration of succinic acid in water. The concentration of succinic acid increases as the partition coefficient values decrease.

k _D_ = 0.05462

slope = −0.11722 = 2k _D_^2^K = 0.0059666888K

K = −19.6457

This equilibrium constant gives the combined reactions constant for both the succinic anhydride formation and succinate ions formation in this system.

The values of the partition coefficient (k _D_) for succinic acid in diethyl ether-water at 30°C and atmospheric pressure were calculated as shown in Table 14.

Table 14 shows the dissolution of succinic acid in diethyl ether and in water. Succinic acid dissolves more in water than in diethyl ether. The partition coefficient values in this system decrease as the concentration of succinic acid in the system increases in water.

Intercept, k _D_ = 0.0427

slope = 2k _D_^2^K = −0.06538

K = −18.1611

K is the reaction equilibrium constant for the formation of succinic anhydride in diethyl ether.

Table 15 presents the values for concentration of succinic acid in water and the partition coefficient values in diethyl ether-water. The concentration of succinic acid in water decreases as the partition coefficient values decrease.

Intercept, k _D_ = −0.00814

slope = 0.01643 = (Kk _D_) ^1/2^

K = α = 0.0332

K is the reaction equilibrium constant represented as α for the ionization reaction for the formation of succinate ions in water of this system.

Using equation (23) a plot of $\frac{C_{X}^{A}}{C_{X}^{*B}}$ (1− α ) vs $C_{X}^{*B}$ (1− α ) will give a straight line where α is the degree of ionization of succinic acid in water.

Thus, (1− α ) = 0.9668

This table shows the effect of ionization on the concentration of succinic acid in water and on the partition coefficient in diethyl ether-water. The partition coefficient decreases as the concentration of succinic acid increases in water.

k _D_ = 0.04122

slope = −0.06514 = 2k _D_^2^K = 0.0033981768K

K = −19.17.

K is the equilibrium constant for the combined reactions of anhydride formation and succinate ions formation in this system.

The values of the partition coefficient (k _D_) for succinic acid in n-hexane-water at 30°C and atmospheric pressure were calculated as shown in Table 17

Table 17 shows the dissolution of succinic acid in n-hexane and in water. Succinic acid dissolves more in water than in n-hexane, and the partition coefficient value increases with the increase in the concentration of succinic acid in the system.

Intercept, k _D_ = −0.04274

slope = 2k _D_^2^K = 0.26286

K = 71.9491

K is the equilibrium constant for the formation of succinic anhydride in n-hexane.

Table 18 relates the concentration of succinic acid in water and the partition coefficient in n-hexane-water. The concentration of succinic acid in water decreases as the partition coefficient values increase.

Intercept, k _D_ = 0.15664

slope = −0.06448 = (Kk _D_) ^1/2^

K = α = 0.0265

K gives the reaction equilibrium constant for the ionization of succinic acid, α , in water in this system.

Using equation (23) a plot of $\frac{C_{X}^{A}}{C_{X}^{*B}}$ (1− α ) vs $C_{X}^{*B}$ (1− α ) will give a straight line, α is the degree of ionization of succinic acid in water.

Thus, (1 − α ) = 0.9735

Table 19 shows the effect of ionization on the concentration of succinic acid in water and on the partition coefficient values in n-hexane-water. The concentration of succinic acid in water increases linearly; the partition coefficient increases too with the increase in concentration of succinic acid in the system.

k _D_ = −0.04161

slope = 0.26278 = 2k _D_^2^K = 0.0034627842K

K = 75.89

This is the equilibrium constant for the combined reactions for the formation of succinic anhydride and succinate ions in this system.

Succinic acid has the partition coefficient (k _D_) values of −0.0562 in carbon tetrachloride-water, 0.0427 in diethyl ether-water and −0.0427 in n-hexane-water, as shown in Table 20. The formation of succinic anhydride occurred in the three systems with the highest value of 71.9491 occurring in the n-hexane-water medium. The ionization reaction in the three systems was relatively low. Carbon tetrachloride-water is the best binary immiscible solvent for succinic acid analyses from the three systems that were investigated.

## Conclusion

This research emphasizes the use of binary immiscible solvents in the extraction of solutes from contaminants and from the ores. Carbon tetrachloride-water is recommended for the analyses of both oxalic acid and succinic acid.

From the results in Table 10, oxalic acid has the highest partition coefficient value of 0.0738, the dimerization constant value of 15.7092, in magnitude, and the ionization constant value of 0.0303. These values show that extraction of oxalic acid in carbon tetrachloride is possible. Additionally, there were interfering dimerization reactions in the carbon tetrachloride, and ionization reactions of the oxalic acid in the water. This is similar to the case of succinic acid with the highest partition coefficient value of 0.0562, in magnitude, and with the dimerization constant value of 18.5655 in magnitude, and ionization constant value of 0.0284. The extraction of succinic acid with carbon tetrachloride is possible.

The reaction conditions of 30°C and atmospheric pressure were used for this research. In future research, we recommend a variety of temperature and pressure conditions are used in order to determine the best temperature for these extractions.

Some challenges were faced in the process of this research, including having a steady network for studies and taking precautionary measures while analyzing the solutes to avoid their excessive inhalations.

Partition coefficient technique is a good analytical parameter for the analyses of materials in binary immiscible solvent. The partition coefficient value resulting from the use of binary immiscible solvent serves as a tool to underscore the efficiency of the partition coefficient technique. Other parameters such as dimerization constant, K, ionization constant, α , also show the degree and efficiency of the process. Partition coefficient technique is an excellent method of purification, and extraction of solutes, from binary immiscible solvents. A high partition coefficient shows that there is dissolution of the solute in the solvent involved, and therefore good separation is assured.

The equation,

$$\frac{C_{X}^{A}}{C_{X}^{*B}}=k_{D}+(x\;{k_{D}}^{x}\frac{K}{m^{m}n^{n}}){}^{\frac{1}{m+n}}{C_{X}^{*B}}^{\left(\frac{x}{m+n}\right)-1}$$

is the general equation for the consideration of the effects of solute interactions in binary immiscible solvent used in the solvent extractions. Thus,

$$\frac{C_{X}^{A}}{C_{X}^{*B}}=k_{D}+\beta {\left(C_{X}^{*B}\right)}^{y}$$

where

$$\beta =x{k_{D}}^{x}{\left(\frac{K}{m^{m}n^{n}}\right)}^{\frac{1}{m+n}}\;$$

and

$$y=\left(\frac{x}{m+n}\right)-1$$

could be used for all types of solute interactions, namely, association, ionization, etc.

Partition coefficient technique is the efficient method for purification, beneficiation, and extraction of materials from the impure state. It is recommended for laboratories, and industries for the analyses of materials in binary immiscible solvents, for purification of impure substances, and for concentration of substances and for the beneficiation of materials from the impure states.

Systems left alone in nature run down to a stage where the observable properties that describe such systems become independent of time. Examples of such systems are:

1 Heat flows from a hot body to a cold body until both bodies have similar temperatures.
2 Matter diffuses from a position of high concentration to a position of low concentration until the concentrations become even.
3 Water runs down the hill
4 A ball dropped on a pavement eventually stops bouncing.
5 A mixture of hydrocarbon gas and air burns to give water and carbon dioxide and later the reaction stops.

In all of these examples above, there is a difference in the property of the system which could be described as property gradient. There is property gradient formed in the manner of temperature gradient from state 1 to state 2 (T _1_ to T _2_), in the manner of concentration gradient, from concentration 1 to concentration 2 (C _1_ to C _2_) etc. These natural processes occur with no loss of energy by the system, they occur of their own accord and are spontaneous and irreversible. All naturally occurring processes always change spontaneously in a direction that leads to equilibrium and the driving force lies at the heart of thermodynamics with special emphasis on increase in entropy. The maximum work a process may perform is the true measure of the driving force that accompanies the process.

The concept of property gradient observed in the natural processes above is created by the use of binary immiscible solvents with differential solubilities of a solute in the solvents. The driving force is defined by the partition coefficient. When a binary immiscible solvent is used as discussed in this work, efficiency of solvent extraction is enhanced.

## Data availability

Zenodo: Comparisons of the effects of solute interactions on partition coefficient, k _D_, in selected binary immiscible solvents: a case of oxalic acid and succinic acid. https://doi.org/10.5281/zenodo.5171412. ^28^

Data are available under the terms of the Creative Commons Attribution 4.0 International license (CC-BY 4.0).

## References

[1] Onyeocha VO Akpan OD Onuchukwu IA : The Dimerization Effects of Some Solutes on the Partition Coefficient (k _D_) in Binary Immiscible Solvents. *Intern. Lett. Chem. Phys. Astrono.* 2018;80:40–52. 10.18052/www.scipress.com/ILCPA.80.40 Reference Source

[2] Onuchukwu AI : *Chemical Thermodynamics for Science Students.* Owerri: FUTO Press; 4th ed. 2016;175.

[3] Rastogi RP Misra RR : *An Introduction to Chemical Thermodynamics.* India: Vikas Publishing House Pvt Ltd; Second Edition. 1980; pp296–300.

[4] Staunton S Geoderma : Sensitivity Analysis of the Distribution Coefficient, k _D_, of Nickel with Changing Soil Chemical Properties. Oct 2004.

[5] Othmer K : Encyclopaedia of Chemical Technology. 1979; Vol,1: pp147. Third Edition.

[6] Morrison RT Boyd RN : *Organic Chemistry.* Sixth Edition;2005;375.

[7] Aston JG Fritz JJ : *Thermodynamics and Statistical Thermodynamics.* New York: John Wiley and Sons Inc;1963;105.

[8] Smith JM Van Ness HC : *Introduction to Chemical Engineering Thermodynamics.* New York: McGraw-hill Book Company; Fifth ed. 1996;225.

[9] Jerry M : *Advanced Organic Chemistry, Reactions, Mechanisms and Structure.* New York: John Wiley and Sons Inc; Second ed 2003;307.

[10] Graham Solomons TW : *Organic Chemistry.* New York: Third Edition;1984;375.

[11] Atkins P Jones L : *Chemistry Molecules, Matter and Change.* New York: Sumanas, Inc., and W. H. Freeman and Company; Third Edition 1997; pp437–463, 490.

[12] Keolopile ZG Ryder MR Gutowski M : Intermolecular Interactions between Molecules in Various Conformational States: The Dimer of Oxalic Acid. *J. Phys. Chem. A.* 2014;118:7385, 7385-7391, June 13, 2014. 10.1021/jp4125638 24923870

[13] Dingrando L Tallman KG Hainen N : *Chemistry Matter and Change.* New York: The McGraw-Hill Companies, Inc.;2005;452–486.

[14] Snell-Hilton : Encyclopaedia of Industrial Chemical Analysis. 1979;4:101.

[15] Onuchukwu AI : *Electrochemical Technology.* Ibadan: Spectrum Books Limited;2008;33–35.

[16] Philips JS Strozak VS Wistrom C : *Chemistry Concepts and Applications.* New York: Glencoe/McGraw-Hill;2005;451–472.

[17] Davis RE Frey R Sarquis M : *Modern Chemistry.* New York: A Harcourt Education Company; Teacher Edition.pp400–425, 498-522.

[18] Atkins P Paula J de : *Physical Chemistry.* New York: W. H. Freeman and Company;2002; pp161–175.

[19] Atkins P : *Physical Chemistry.* New York: W. H. Freeman and Company; Fifth ed 1993;213–231.

[20] Fong P : *Foundation of Thermodynamics.* New York: Oxford University Press;1963;73.

[21] Manhan BH : *Elementary Chemical Thermodynamics.* Third ed. New York: W. A. Benjamin Inc;1963;207.

[22] Lambert J Muir TA : *Practical Chemistry.* London: Heinemann Educational Book Ltd; Third ed. 1973;273.

[23] Mei PR Moreira SP Cortes ADS : Determination of the effective distribution coefficient for silicon impurities. *J. Renew. Sustain. Ener.* 2012;4:043118. 10.1063/1.4739759

[24] Tetko IV Poda GI : Application of ALOGPS 2.1 to predict log k _D_ Distribution Coefficient for Pfizer proprietary Compounds. *J. Med. Chem.* 2004;47:5601–5604. 10.1021/jm049509l 15509156

[25] Kodolikar-Kulkarni S Bhatkhande D : Prediction of effectiveness of solvent using distribution coefficient calculation in solvent extraction with the help of Hansen solubility parameters. 2020.

[26] Barnes N Gramajo de Doz M Solimo HN : Liquid-liquid extraction of oxalic acid from aqueous solutions with tributyl phosphate and a mixed solvent at 303.15K. 1999.

[27] Laboratory Chemicals and Biochemical (BDH) 1982; pp220. Export Edition.

[28] Onyeocha VO : Comparisons of the effects of solute interactions on partition coefficient, kD, in selected binary immiscible solvents: a case of oxalic acid and succinic acid. 2021. 10.5281/zenodo.5171412 PMC10521093 37767079

[29] Nicholas P : *Cheremisinoff, Motasem B. Haddadin, Beyond Compliance.* Gulf Publishing Company;2006. 10.1016/B978-0-9765113-9-7.50005-5

