# Supplementary material for: Comparisons of the effects of solute interactions on partition coefficient, k D, in selected binary immiscible solvents: a case of oxalic acid and succinic acid
Source: F1000Res. 2022 Jan 19;11:63. [Version 1] doi: 10.12688/f1000research.55200.1 (PMC10521093; doi:10.12688/f1000research.55200.1)
Supplement: Peer review history

# Reviewer report: Reviewer response for version 1

**DOI:** 10.5256/f1000research.58757.r161096

**Published:** 2023-03-22

**Authors:** Ana C. Gomez Marigliano

**Affiliations:** 1 Universidad Nacional de Tucumán, Tucumán, Argentina

This is a work that I consider adequate, due to the number of tests carried out and the possible applications at an environmental level that derive from the results. However, I have a few recommendations.

1. In page 3 – line 1 and line 6, write "immiscible solvent binary system" instead of “binary solvent system”.
2. In page 3, after equation 4: only in A?
3. The solvent strength really needs to be much greater or much less than one to be efficient...check the equation 15.
4. Must give purity of reagents and uncertainties of the results obtained and also the instruments used to measure temperature and pressure.
5. When you made the composition measurements of each phase. How are you sure that it reached equilibrium?
6. Results must be given with uncertainty.

Is the work clearly and accurately presented and does it cite the current literature?

Partly

If applicable, is the statistical analysis and its interpretation appropriate?

Not applicable

Are all the source data underlying the results available to ensure full reproducibility?

Yes

Is the study design appropriate and is the work technically sound?

Partly

Are the conclusions drawn adequately supported by the results?

Yes

Are sufficient details of methods and analysis provided to allow replication by others?

Yes

Reviewer Expertise:

thermodymanics

I confirm that I have read this submission and believe that I have an appropriate level of expertise to confirm that it is of an acceptable scientific standard.

# Reviewer report: Reviewer response for version 1

**DOI:** 10.5256/f1000research.58757.r120391

**Published:** 2022-04-21

**Authors:** Shilpa Prasad Kodolikar

**Affiliations:** 1 Department of Chemical Engineering, MIT World Peace University, Pune, Maharashtra, India

This manuscript describes the methodology for partition coefficient predictions based on molecular distribution of solutes. The overall approach is well appreciated.

 Some redeeming features are the molecular distribution approach selection for partition coefficient technique, well  defined objective of all methodology, successful implementation and application of technique to industrial systems.

 Some points to revisit can be including some latest literature, post 2018, reconstructing some sentences [pg 5 para 1 line 2]. Overall the paper is good.

Is the work clearly and accurately presented and does it cite the current literature?

Yes

If applicable, is the statistical analysis and its interpretation appropriate?

Yes

Are all the source data underlying the results available to ensure full reproducibility?

Yes

Is the study design appropriate and is the work technically sound?

Yes

Are the conclusions drawn adequately supported by the results?

Yes

Are sufficient details of methods and analysis provided to allow replication by others?

Yes

Reviewer Expertise:

Liquid separations, process design, mathematical modeling and simulation, ionic liquids

I confirm that I have read this submission and believe that I have an appropriate level of expertise to confirm that it is of an acceptable scientific standard.
